# Electrochemical Bromination of Glycals

**DOI:** 10.3389/fchem.2021.796690

**Published:** 2021-12-23

**Authors:** Zhao-Xiang Luo, Miao Liu, Tian Li, De-Cai Xiong, Xin-Shan Ye

**Affiliations:** ^1^ State Key Laboratory of Natural and Biomimetic Drugs, School of Pharmaceutical Sciences, Peking University, Beijing, China; ^2^ State Key Laboratory of Pharmaceutical Biotechnology, School of Life Sciences, Nanjing University, Nanjing, China

**Keywords:** electrochemistry, bromination, glycals, 2-bromoglycals, cross-coupling, ferrier rearrangement

## Abstract

Herein, the convenient one-step electrochemical bromination of glycals using Bu_4_NBr as the brominating source under metal-catalyst-free and oxidant-free reaction conditions was described. A series of 2-bromoglycals bearing different electron-withdrawing or electron-donating protective groups were successfully synthesized in moderate to excellent yields. The coupling of tri-*O*-benzyl-2-bromogalactal with phenylacetylene, potassium phenyltrifluoroborate, or a 6-OH acceptor was achieved to afford 2C-branched carbohydrates and disaccharides via Sonogashira coupling, Suzuki coupling, and Ferrier rearrangement reactions with high efficiency. The radical trapping and cyclic voltammetry experiments indicated that bromine radicals may be involved in the reaction process.

## Introduction

Carbohydrates mainly exist in the form of glycoconjugates, polysaccharides, oligosaccharides, and monosaccharides and play a pivotal role in a broad range of important biological processes including cell proliferation, host–pathogen interactions, cell adhesion, hormone function, and the immune response (Kiessling and Kraft, 2013; Wang et al., 2020). Chemical synthesis can afford both naturally occurring important carbohydrates and biologically active carbohydrate mimetics in sufficient quantities, providing a powerful tool to understand the biological functions of carbohydrates (Muthana et al., 2009; Panza et al., 2018; Li and Ye, 2020).

**SCHEME 1:**
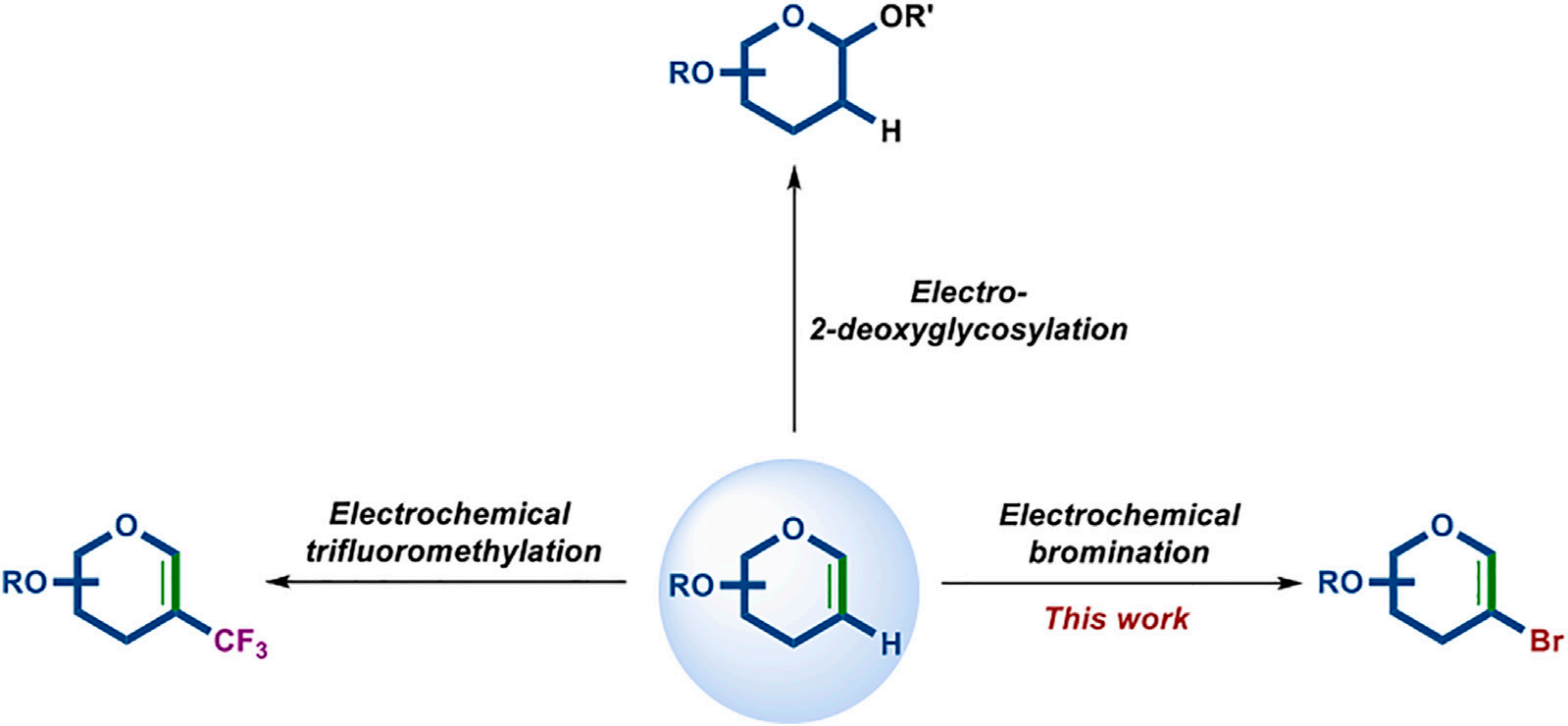
Electrochemical transformation of glycals.

Organic electrosynthesis is of current interest as one of the most promising methods for the efficient, sustainable, and green synthesis of medicinally significant compounds (Francke and Little, 2014; Horn et al., 2016; Liang and Zeng, 2020; Meyer et al., 2020; Yuan et al., 2021). In recent years, the electrochemical synthesis of oligosaccharides has been successfully demonstrated through the activation of different types of glycosyl donors, such as thio-, seleno-, and telluro-glycosides (Nokami et al., 2015; Manmode et al., 2018; Zhang et al., 2020). In addition, our group has been involved in the electrochemical transformation of glycals to obtain significant synthetic carbohydrate compounds (Liu et al., 2020; Liu et al., 2021). Along with the use of MnBr_2_ as the redox mediator, the electrochemical trifluoromethylation of glycals has been realized (Liu et al., 2021). In the exploration of this reaction, we found that 2-bromoglycals could also be isolated when the equivalent of Bu_4_NBr was added. Inspired by this surprising result, we turned our focus to the electrochemical bromination of glycals (Scheme 1).

Over the past few decades, 2-bromoglycals have been widely employed as important synthons in combination with metal-catalyzed cross-coupling reactions to access 2C-branched carbohydrates and their analogs (Leibeling et al., 2010a; Leibeling et al., 2010b; Leibeling and Werz, 2012; Dharuman and Vankar, 2014; Martin et al., 2015). Due to the importance of 2-bromoglycals, the development of a novel, practical, and environmentally friendly method for the synthesis of 2-bromoglycals is still of high interest. The most common way to obtain 2-bromoglycals consists of two steps using Br_2_, which is toxic and unstable, as the brominating source (Leibeling et al., 2010a). An alternative approach involves the one-step synthesis of 2-bromoglycals from glycals using *N*-bromosuccinimide and silver nitrate (Dharuman and Vankar, 2014). We herein report a one-step electrochemical bromination of glycals using commercially available, stable, and safe Bu_4_NBr as the bromine source in an undivided cell under metal-catalyst-free and oxidant-free reaction conditions.

## Results and Discussion

Initially, we began our investigation with 6-*O*-benzyl-3,4-dideoxy-glycal **1a** as the model substrate for electrochemical bromination using Pt as the anode and cathode in an undivided cell. Unfortunately, no desired 2-bromo-3,4-dideoxy-glycal **3a** was detected using KBr (Ye and Shreeve, 2004; Zhao and Lu, 2018) or NaBr (Alberto et al., 2014) as the “Br” reagent (Table 1, entries 1 and 2). To our delight, the product **3a** could be obtained in 10% isolated yield when **1a** was treated with 1.5 equiv of Bu_4_NBr (Yoshimitsu et al., 2009; Kamon et al., 2012) in dry CH_3_CN at room temperature under a constant electric current of 2 mA (Table 1, entry 3). The yield was increased slightly when the reaction temperature was increased to 50°C (Table 1, entry 4). Further raising the temperature to 75°C was beneficial to this transformation, leading to the formation of **3a** in 35% yield (Table 1, entry 5), and the yield could be increased to 40% when the amount of Bu_4_NBr was increased to 2.0 equiv (Table 1, entry 6). It was found that the addition of base, such as K_2_CO_3_ or Na_2_CO_3_, could further improve the reaction yield (Table 1, entries 7 and 8). Surprisingly, when the bromination reaction was conducted with NaSO_2_CF_3_ as the additive, the desired product **3a** was isolated in 67% yield (Table 1, entry 9). Altering the amount of NaSO_2_CF_3_ to 2.0 equiv led to an increased yield of 82% (Table 1, entry 10). Comparatively, when other solvents such as CH_3_CN/H_2_O (3:1), 1,2-dimethoxyethane, or ClCH_2_CH_2_Cl, were used instead of dry CH_3_CN, lower yields were achieved (Table 1, entries 11–13). Extensive screening experiments revealed that either changing electrode materials or modifying the reaction current were not effective for improving the yield of **3a** (Table 1, entries 14–18). Moreover, the yield of **3a** was decreased drastically when the electrochemical bromination reaction was performed in an air atmosphere (Table 1, entry 19). Finally, the control experiment confirmed that the role of electricity was essential, as the reaction could not proceed in the absence of an electric current (Table 1, entry 20).

**TABLE 1:** Optimization of reaction conditions^a^.

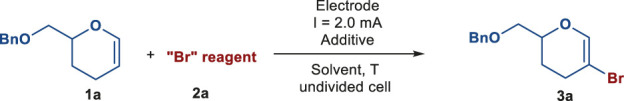						
Entry	Electrode	“Br” reagent	Additive	Solvent	T (°C)	Yield (%)^b^
1	Pt (+)/Pt (−)	KBr (1.5 equiv)	—	CH_3_CN	Rt	0 (0)
2	Pt (+)/Pt (−)	NaBr (1.5 equiv)	—	CH_3_CN	Rt	0 (0)
3	Pt (+)/Pt (−)	Bu_4_NBr (1.5 equiv)	—	CH_3_CN	Rt	10 (0)
4	Pt (+)/Pt (−)	Bu_4_NBr (1.5 equiv)	—	CH_3_CN	50°C	18 (14)
5	Pt (+)/Pt (−)	Bu_4_NBr (1.5 equiv)	—	CH_3_CN	75°C	35 (10)
6	Pt (+)/Pt (−)	Bu_4_NBr (2.0 equiv)	—	CH_3_CN	75°C	40 (13)
7	Pt (+)/Pt (−)	Bu_4_NBr (2.0 equiv)	K_2_CO_3_ (1.2 equiv)	CH_3_CN	75°C	43 (4)
8	Pt (+)/Pt (−)	Bu_4_NBr (2.0 equiv)	Na_2_CO_3_ (1.2 equiv)	CH_3_CN	75°C	54 (6)
9	Pt (+)/Pt (−)	Bu_4_NBr (2.0 equiv)	NaSO_2_CF_3_ (1.2 equiv)	CH_3_CN	75°C	67 (0)
10	Pt (+)/Pt (−)	Bu_4_NBr (2.0 equiv)	NaSO_2_CF_3_ (2.0 equiv)	CH_3_CN	75°C	82 (0)
11	Pt (+)/Pt (−)	Bu_4_NBr (2.0 equiv)	NaSO_2_CF_3_ (2.0 equiv)	CH_3_CN/H_2_O (3/1)	75°C	15 (0)
12	Pt (+)/Pt (−)	Bu_4_NBr (2.0 equiv)	NaSO_2_CF_3_ (2.0 equiv)	1,2-Dimethoxyethane	75°C	46 (7)
13	Pt (+)/Pt (−)	Bu_4_NBr (2.0 equiv)	NaSO_2_CF_3_ (2.0 equiv)	ClCH_2_CH_2_Cl	75°C	Trace (0)
14	C (+)/Pt (−)	Bu_4_NBr (2.0 equiv)	NaSO_2_CF_3_ (2.0 equiv)	CH_3_CN	75°C	71 (0)
15	Pt (+)/C (−)	Bu_4_NBr (2.0 equiv)	NaSO_2_CF_3_ (2.0 equiv)	CH_3_CN	75°C	32 (3)
16	C (+)/C (−)	Bu_4_NBr (2.0 equiv)	NaSO_2_CF_3_ (2.0 equiv)	CH_3_CN	75°C	33 (7)
17^c^	Pt (+)/Pt (−)	Bu_4_NBr (2.0 equiv)	NaSO_2_CF_3_ (2.0 equiv)	CH_3_CN	75°C	58 (0)
18^d^	Pt (+)/Pt (−)	Bu_4_NBr (2.0 equiv)	NaSO_2_CF_3_ (2.0 equiv)	CH_3_CN	75°C	54 (0)
19^e^	Pt (+)/Pt (−)	Bu_4_NBr (2.0 equiv)	NaSO_2_CF_3_ (2.0 equiv)	CH_3_CN	75°C	Trace (0)
20^f^	Pt (+)/Pt (−)	Bu_4_NBr (2.0 equiv)	NaSO_2_CF_3_ (2.0 equiv)	CH_3_CN	75°C	NR (97)

a Reaction conditions: **1a** (0.05 mmol), “Br” reagent, Additive, Solvent (4.0 ml), Electrode, constant current = 2.0 mA, T, 4 h, in an undivided cell, under an argon atmosphere.

b Yield of the isolated product, the yield of recovered starting material was represented in the parentheses.

c I = 1.0 mA

d I = 3.0 mA

e Under an air atmosphere.

f No electricity.

With the optimal reaction conditions in hand, we then evaluated the substrate scope of the electrochemical bromination of various types of glycals with Bu_4_NBr (Table 2). First, 3,4-dideoxy-glycals with electron-withdrawing groups were examined. Substrates with an acetyl or benzoyl group provided the respective brominated products **3b** and **3c** in good yields. In addition, benzyl (Bn), *p*-methoxybenzyl (PMB), *tert*-butyldimethylsilyl (TBS), and methyl (Me) substituted glucals could also be converted into the corresponding products **3d**–**g**. Similarly, galactals bearing Bn, PMB, or TBS groups were found to be amenable to the electrochemical reaction, providing the desired products **3h–j** in 56–73% yields. And the scalability of this electrochemical bromination was further showed by an efficient conversion of compound **1h** on a 250 mg scale in 57% isolated yield. Peracetylated and perbenzoylated galactals could also undergo the electrochemical transformation to afford 2-bromo-galactals **3k**–**l**, albeit in slightly low yields. Notably, bromination of peracetylated L-rhamnal proceeded smoothly to deliver compound **3m** in 89% yield. Under the same conditions, the L-rhamnal, L-arabinal, and D-xylal equipped with Bn or PMB groups were also able to furnish the corresponding brominated products **3n**–**s** in moderate to excellent yields. Furthermore, benzylated lactal **1t** underwent this reaction to afford the desired product **3t** in 72% yield.

**TABLE 2:** Substrate scope of glycals^a^ ^,^ ^b^ ^,^ ^c^ ^,^ ^d^ ^,^ ^e^.

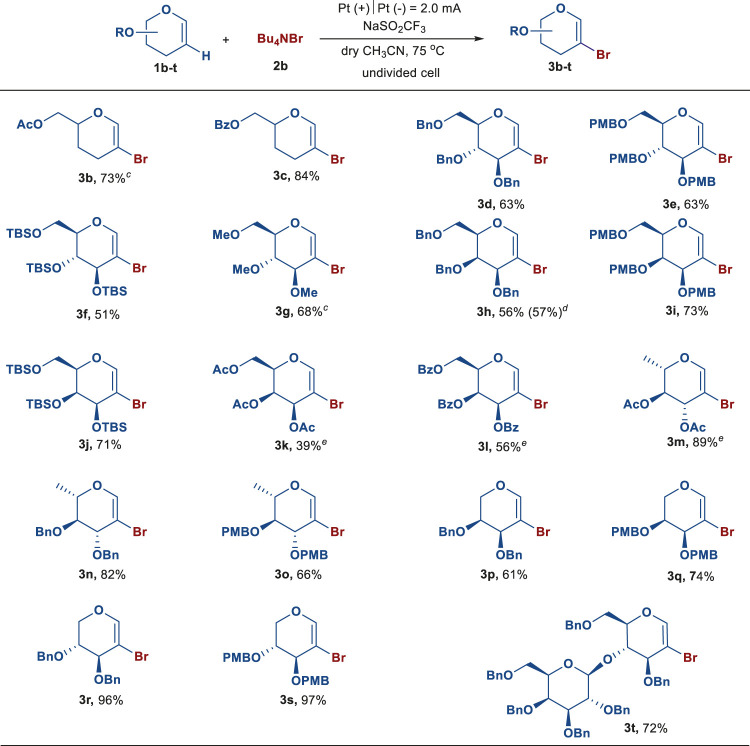

a Reaction conditions: glycals (0.05 mmol), NaSO_2_CF_3_ (0.10 mmol, 2.0 equiv), Bu_4_NBr (0.10 mmol, 2.0 equiv), dry CH_3_CN (4.0 ml) in an undivided cell with Pt as the anode and cathode, constant current = 2.0 mA, 75°C, under argon atmosphere, 4 h.

b Yield of the isolated product.

c glycals (0.10 mmol), NaSO_2_CF_3_ (0.20 mmol, 2.0 equiv), Bu_4_NBr (0.30 mmol, 3.0 equiv), dry CH_3_CN (5.0 ml) in an undivided cell with Pt as the anode and cathode, constant current = 2.0 mA, 75°C, under argon atmosphere, 6 h.

d glycals (0.60 mmol), NaSO_2_CF_3_ (1.20 mmol, 2.0 equiv), Bu_4_NBr (1.20 mmol, 2.0 equiv), dry CH_3_CN (50.0 ml) in an undivided cell with Pt as the anode and cathode, constant current = 2.0 mA, 75°C, under argon atmosphere, 30 h.

e glycals (0.05 mmol), NaSO_2_CF_3_ (0.10 mmol, 2.0 equiv), Bu_4_NBr (0.15 mmol, 3.0 equiv), dry CH_3_CN (4.0 ml) in an undivided cell with Pt as the anode and cathode, constant current = 2.0 mA, 75°C, under argon atmosphere, 6 h.

**SCHEME 2:**
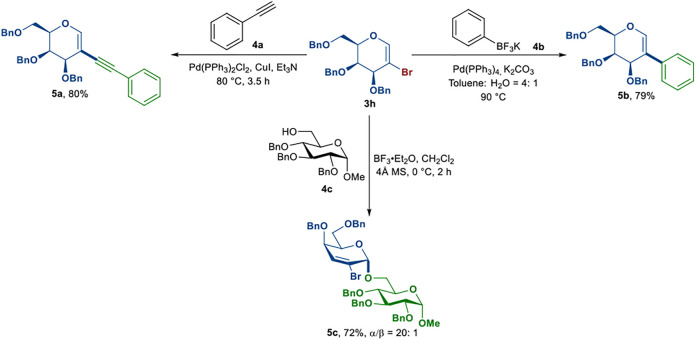
Reaction of 2-bromogalactal **(3h)** with different substrates **(4a–c)**.

To demonstrate the potential applicability of 2-bromoglycals, the reaction of 2-bromogalactal **3h** with different substrates was carried out (Scheme 2). First, we explored the utility of 2-bromogalactal **3h** in the synthesis of 2C-substituted carbohydrates, which exist in many natural products (Yin and Linker, 2012; Dubbu et al., 2018; Darbem et al., 2020). Compound **3h** reacted with phenylacetylene **4a** in the presence of Pd(PPh_3_)_2_Cl_2_, CuI and Et_3_N to afford the coupled product **5a** in 80% yield (Koester and Werz, 2012). The reaction of **3h** with potassium phenyltrifluoroborate **4b** also proceeded smoothly to provide the corresponding product **5b** in 79% yield (Molander and Fumagalli, 2006). Moreover, disaccharide **5c** was successfully synthesized in the promotion of BF_3_·Et_2_O in 72% yield with excellent α-selectivity via the Ferrier rearrangement reaction (Dharuman et al., 2013; Wang et al., 2019).

**SCHEME 3:**
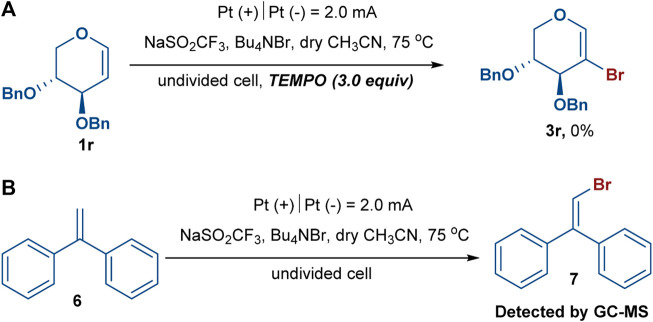
In the radical trapping experiments, experiment **(A)** was performed with glycal (0.05 mmol) and TEMPO (0.15 mmol, 3.0 equiv) under standard reaction condition: NaSO2CF3 (0.10 mmol, 2.0 equiv), Bu4NBr (0.10 mmol, 2.0 equiv), dry CH3CN (4.0 ml) in an undivided cell with Pt as the anode and cathode, constant current = 2.0 mA, 75 oC, under argon atmosphere, 4 h. Besides, experiment **(B)** was performed with 1,1-diphenylethylene (0.15 mmol) under standard reaction condition as well.

**FIGURE 1:**
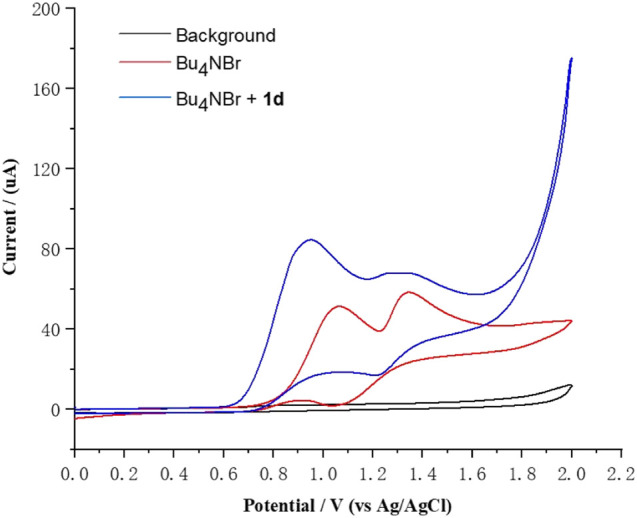
Cyclic voltammetry measurements of Bu_4_NBr and **1d**. Conditions: glassy carbon disk electrode (diameter is 3.0 mm, PTFE shroud) as the working electrode, platinum wire as the counter electrode, Ag/AgCl electrode (3.5 M KCl solution) as the reference electrode, Bu_4_NOTf (0.10 M in MeCN), under an argon atmosphere, cyclic voltammogram at 0.05 V s^−1^ with Bu_4_NBr (5 mM) or Bu_4_NBr (5 mM) and **1d** (5 mM).

To gain insight into the mechanism of this electrochemical bromination, radical trapping experiments were performed. As expected, the reaction was completely shut down when 3.0 equiv of the radical scavenger 2,2,6,6-tetramethylpiperidine-1-oxyl (TEMPO) was added under the standard reaction conditions, indicating that radical chemistry was likely involved in the reaction (Scheme 3A) (Makai et al., 2020; Wang et al., 2019). Furthermore, another experiment using 1,1-diphenylethylene (**6**) was also conducted under the standard reaction conditions, and (2-bromoethene-1,1-diyl)dibenzene (**7**) was successfully detected in the GC–MS, confirming the participation of the bromine radical in the reaction system (Scheme 3B) (Chen et al., 2020; Kale et al., 2021). We observed that the reaction mixture gradually turned brown during the reaction process, indicating that Br_2_ might be generated. The 2-bromoglycal **3h** was resubmitted to the standard reaction conditions for 20 h with the recovery of **3h** in a 78% yield. In addition, cyclic voltammetry experiments were carried out to investigate the redox behavior of the reaction. The cyclic voltammetry measurements of Bu_4_NBr indicated two obvious oxidative peaks at 1.07 and 1.37 V (vs. Ag/AgCl) (Figure 1, red curve), which likely corresponded to Br_3_^–^/Br^–^ and Br_3_^–^/Br_2_ redox couples, respectively (Damljanovic et al., 2011; Bennett et al., 2016; Kang et al., 2016). The first oxidative peak was associated with the oxidation of Br^–^ to the bromine radical. The bromine radical then integrated into Br_2_, which could combine with Br^–^ to form Br_3_^–^. The second oxidative peak was attributed to the oxidation of Br_3_^–^ to Br_2_. An increase in the oxidative peak current was observed when Bu_4_NBr and **1d** were combined, which was attributed to a catalytic current, resulting from the chemical reaction of the electrochemically-generated Br_2_ and glucal **1d** (Figure 1, blue curve).

### Mechanism

**SCHEME 4:**
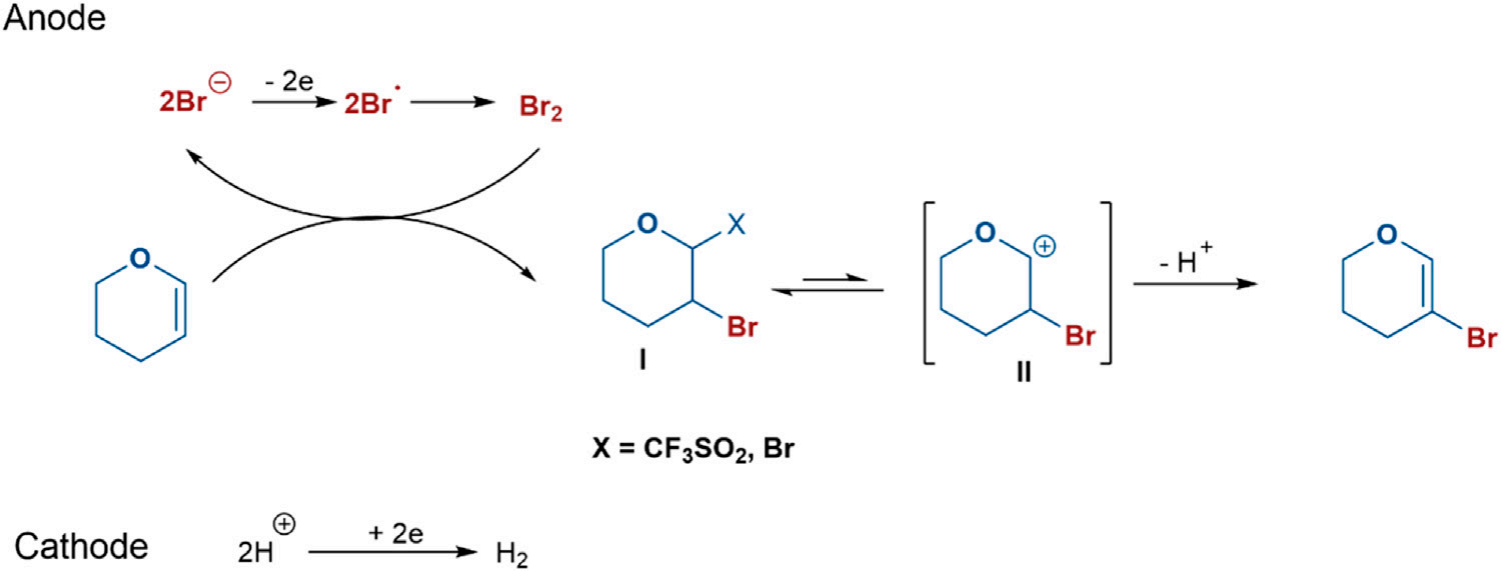
Plausible reaction mechanism for the electrochemical bromination of glycals.

Based on the above results and previous reports (Yuan et al., 2019; Gou et al., 2021; Wu et al., 2021), a plausible reaction mechanism for the electrochemical bromination of glycals was depicted in Scheme 4. A bromine anion was oxidized to the bromine radical on the anode and subsequently molecular Br_2_. This was then attacked by glycal to yield the intermediate **I**. Finally, the brominated product was obtained by the deprotonation of **II**, which would be stabilized by acetonitrile. NaSO_2_CF_3_ might be used as the electrolyte to increase the conductivity of the reaction solution and a proton scavenger to generate strong acid for cathode reduction; besides, it may be an anion to stabilize the glycosyl cation. At the same time, H^+^ was reduced to produce hydrogen on the cathode.

## Conclusion

In summary, we performed the one-step electrochemical bromination of various glycals with electron-withdrawing and electron-donating protective groups using commercially available, nontoxic Bu_4_NBr as the brominating source under metal-catalyst-free and oxidant-free reaction conditions. The synthetic applicability of 2-bromoglycals has been demonstrated by providing the corresponding 2C-substituted carbohydrates and disaccharides via palladium-catalyzed cross-coupling reactions and the Ferrier rearrangement reaction. The readily available substrates and ease of handling make this methodology a practical tool to access diversified brominating synthons for the preparation of biologically relevant carbohydrates.

## Data Availability

The original contributions presented in the study are included in the article/Supplementary Material, further inquiries can be directed to the corresponding authors.
