# Supplementary material for: Electrochemical Bromination of Glycals
Source: Front Chem. 2021 Dec 23;9:796690. doi: 10.3389/fchem.2021.796690 (PMC8732377; doi:10.3389/fchem.2021.796690)
Supplement: Supplementary file 1 [file DataSheet1.docx]

Supplementary Material

Electrochemical Bromination of Glycals

Zhao-Xiang Luo^1†^, Miao Liu^1†^, Tian Li^1^, De-Cai Xiong^1,2*^ and Xin-Shan Ye^1*^

^1^State Key Laboratory of Natural and Biomimetic Drugs, School of Pharmaceutical Sciences, Peking University, Beijing, China

^2^State Key Laboratory of Pharmaceutical Biotechnology, School of Life Sciences, Nanjing University, Nanjing, Jiangsu, China

*** Correspondence:**decai@bjmu.edu.cn; xinshan@bjmu.edu.cn

# Table of Contents

1. General methods.................................................................................................................................3

# 2. General procedures for the electrochemical bromination...................................................................3

3. The synthesis of compounds **5a-c**.......................................................................................................9

4. References.........................................................................................................................................11

5. ^1^H and ^13^C NMR Spectra..................................................................................................................12

1. **General methods**

All the reagents and solvents were purchased from commercial suppliers and used directly without further purification unless otherwise stated. CH_2_Cl_2_ was distilled over calcium hydride prior to use. Dry CH_3_CN was bought from J&K Scientific in China. Visualization on thin-layer chromatography (TLC) was achieved by use of UV light (254 nm), a solution of concentrated sulfuric acid (5 mL) in CH_3_CH_2_OH (95 mL) or a solution of (NH_4_)_6_Mo_7_O_24_•4H_2_O (12.00 g, 9.7 mmol) and Ce(NH_4_)_2_(NO_3_)_6_ (0.25 g, 0.45 mmol) in sulfuric acid (5%, 250 mL). Column chromatography was conducted on silica gel (200-300 mesh). NMR spectra were recorded using CDCl_3_ as a solvent on a Bruker AV 400 or Bruker AV 600 at 400 MHz for ^1^H NMR, 101 or 151 MHz for ^13^C NMR, respectively. Chemical shifts are reported in ppm and coupling constants of ^1^H NMR are expressed in Hz. The multiplicity of data for ^1^H NMR was recorded as follows: s = singlet, d = doublet, t = triplet, q = quartet, m = multiplet, dd = doublet of doublets, and br = broad. High-resolution mass spectra data was obtained using a Waters Xevo G2 Q-TOF mass spectrometer. The electrodes were bought from Xuzhou Xinke Instrument Corp. The potentiostats were bought from Taobao.com, HYELEC 3005B. Cyclic voltammetries were carried on the Metronm Autolab.

1. **General procedures for the electrochemical bromination**

**General procedure A:** To an oven-dried three-neck flask charged with a stir bar was added glycal (0.05 mmol), Bu_4_NBr (0.10 mmol, 32.2 mg), NaSO_2_CF_3_ (0.10 mmol, 15.6 mg) and dry CH_3_CN (4.0 mL). The flask was equipped with two Pt electrodes (2.0 × 1.5 × 0.01 cm^3^) and sealed with rubber stoppers. The reaction mixture was degassed, filled with argon and electrolyzed at 75 ^o^C under constant current of 2.0 mA for 4 h. After the reaction was completed, the reaction mixture was concentrated under reduced pressure. The crude product was eluted by flash column chromatography (petroleum ether/ethyl acetate) to afford the desired product.

**General procedure B:** To an oven-dried three-neck flask charged with a stir bar was added glycal (0.10 mmol), Bu_4_NBr (0.30 mmol, 96.6 mg), NaSO_2_CF_3_ (0.20 mmol, 31.2 mg) and dry CH_3_CN (5.0 mL). The flask was equipped with two Pt electrodes (2.0 × 1.5 × 0.01 cm^3^) and sealed with rubber stoppers. The reaction mixture was degassed, filled with argon and electrolyzed at 75 ^o^C under constant current of 2.0 mA for 6 h. After the reaction was completed, the reaction mixture was concentrated under reduced pressure. The crude product was eluted by flash column chromatography (petroleum ether/ethyl acetate) to afford the desired product.

**General procedure C:** To an oven-dried three-neck flask charged with a stir bar was added glycal (0.05 mmol), Bu_4_NBr (0.15 mmol, 48.4 mg), NaSO_2_CF_3_ (0.10 mmol, 15.6 mg) and dry CH_3_CN (4.0 mL). The flask was equipped with two Pt electrodes (2.0 × 1.5 × 0.01 cm^3^) and sealed with rubber stoppers. The reaction mixture was degassed, filled with argon and electrolyzed at 75 ^o^C under constant current of 2.0 mA for 4 h. After the reaction was completed, the reaction mixture was concentrated under reduced pressure. The crude product was eluted by flash column chromatography (petroleum ether/ethyl acetate) to afford the desired product.

**2-Benzyloxymethyl-5-bromo-3,4-dihydro-2*H*-pyran (3a).** Compound **3a** was prepared as yellow oil (11.6 mg, 82%) from compound **1a** (0.05 mmol, 10.2 mg) after flash column chromatography (petroleum ether: ethyl acetate = 15: 1, v/v) following the general procedure A of the electrochemical bromination. **^1^H NMR** (400 MHz, CDCl_3_) δ 7.38 – 7.27 (m, 5H), 6.65 (t, *J* = 1.7 Hz, 1H), 4.60 (d, *J* = 12.1 Hz, 1H), 4.56 (d, *J* = 12.1 Hz, 1H), 4.06 – 4.00 (m, 1H), 3.63 – 3.49 (m, 2H), 2.55 – 2.46 (m, 1H), 2.37 – 2.30 (m, 1H), 2.00 – 1.93 (m, 1H), 1.91 – 1.83 (m, 1H). **^13^C NMR** (101 MHz, CDCl_3_) δ 142.8, 137.9, 128.4, 127.7, 98.8, 73.7, 73.5, 71.7, 29.1, 25.9. **HRMS** (ESI) Calculated for C_13_H_16_BrO_2_ [M+H]^+^: 283.0334 Found: 283.0330.

**2-Acetoxymethyl-5-bromo-3,4-dihydro-2*H*-pyran (3b).** Compound **3b** was prepared as yellow oil (17.1 mg, 73%) from compound **1b** (0.10 mmol, 15.6 mg) after flash column chromatography (petroleum ether: ethyl acetate = 12: 1, v/v) following the general procedure B of the electrochemical bromination. **^1^H NMR** (400 MHz, CDCl_3_) δ 6.64 (t, *J* = 1.7 Hz, 1H), 4.23 – 4.11 (m, 2H), 4.08 – 4.03 (m, 1H), 2.57 – 2.48 (m, 1H), 2.40 – 2.33 (m, 1H), 2.10 (s, 3H), 2.00 – 1.93 (m, 1H), 1.92 – 1.81 (m, 1H). **^13^C NMR** (151 MHz, CDCl_3_) δ 170.8, 142.5, 98.9, 72.3, 65.5, 28.9, 25.5, 20.8. **HRMS** (ESI) Calculated for C_8_H_12_BrO_3_ [M+H]^+^: 234.9970 Found: 234.9997.

**2-Benzoylmethyl-5-bromo-3,4-dihydro-2*H*-pyran (3c).** Compound **3c** was prepared as yellow oil (12.4 mg, 84%) from compound **1c** (0.05 mmol, 10.9 mg) after flash column chromatography (petroleum ether: ethyl acetate = 20: 1, v/v) following the general procedure A of the electrochemical bromination. **^1^H NMR** (400 MHz, CDCl_3_) δ 8.07 – 8.05 (m, 2H), 7.60 – 7.56 (m, 1H), 7.48 – 7.42 (m, 2H), 6.67 (t, *J* = 1.7 Hz, 1H), 4.48 – 4.36 (m, 2H), 4.26 – 4.15 (m, 1H), 2.63 – 2.52 (m, 1H), 2.44 – 2.37 (m, 1H), 2.10 – 2.03 (m, 1H), 2.01 – 1.89 (m, 1H). **^13^C NMR** (101 MHz, CDCl_3_) δ 166.3, 142.6, 133.2, 129.7, 129.7, 128.4, 98.8, 72.4, 65.8, 28.9, 25.6. **HRMS** (ESI) Calculated for C_13_H_14_BrO_3_ [M+H]^+^: 297.0126 Found: 297.0122.

**2-Bromo-3,4,6-tri-*O*-benzyl-D-glucal (3d).** Compound **3d** was prepared as yellow oil (15.5 mg, 63%) from compound **1d** (0.05 mmol, 20.8 mg) after flash column chromatography (petroleum ether: ethyl acetate = 20: 1, v/v) following the general procedure A of the electrochemical bromination. **^1^H NMR** (400 MHz, CDCl_3_) δ 7.36 – 7.22 (m, 15H), 6.70 (s, 1H), 4.68 (t, *J* = 11.4 Hz, 2H), 4.59 (dd, *J* = 11.4, 6.4 Hz, 2H), 4.52 (s, 2H), 4.31 – 4.27 (m, 1H), 4.12 (d, *J* = 4.4 Hz, 1H), 3.96 (dd, *J* = 5.9, 4.4 Hz, 1H), 3.78 (dd, *J* = 10.7, 5.9 Hz, 1H), 3.72 – 3.66 (m, 1H). The ^1^H NMR data are consistent with those reported (Dharuman and Vankar, 2014).

**2-Bromo-3,4,6-tri-*O*-(*p*-methoxybenzyl)-D-glucal (3e).** Compound **3e** was prepared as yellow oil (18.4 mg, 63%) from compound **1e** (0.05 mmol, 25.3 mg) after flash column chromatography (petroleum ether: ethyl acetate = 5: 1, v/v) following the general procedure A of the electrochemical bromination. **^1^H NMR** (400 MHz, CDCl_3_) δ 7.27 – 7.14 (m, 6H), 6.89 – 6.83 (m, 6H), 6.67 (d, *J* = 0.8 Hz, 1H), 4.65 – 4.43 (m, 6H), 4.26 – 4.20 (m, 1H), 4.08 – 4.06 (m, 1H), 3.90 (dd, *J* = 6.1, 4.5 Hz, 1H), 3.80 (s, 9H), 3.72 (dd, *J* = 10.7, 5.9 Hz, 1H), 3.64 (dd, *J* = 10.7, 4.0 Hz, 1H). **^13^C NMR** (101 MHz, CDCl_3_) δ 159.4, 159.4, 159.3, 144.1, 129.9, 129.7, 129.6, 129.5, 129.4, 113.9, 113.8, 98.8, 77.1, 76.5, 73.7, 73.1, 72.5, 72.0, 67.5, 55.3, 55.3, 55.3. **HRMS** (ESI) Calculated for C_30_H_37_NBrO_7_ [M+NH_4_]^+^: 602.1753 Found: 602.1760.

**2-Bromo-3,4,6-tri-*O*-(*tert*-butyldimethylsilyl)-D-glucal (3f).** Compound **3f** was prepared as yellow oil (14.5 mg, 51%) from compound **1f** (0.05 mmol, 24.5 mg) after flash column chromatography (petroleum ether: ethyl acetate = 15: 1, v/v) following the general procedure A of the electrochemical bromination. **^1^H NMR** (400 MHz, CDCl_3_) δ 6.58 (s, 1H), 4.14 – 4.10 (m, 1H), 3.98 – 3.88 (m, 2H), 3.85 (t, *J* = 2.1 Hz, 1H), 3.73 (dd, *J* = 11.5, 4.6 Hz, 1H), 0.90 (s, 9H), 0.89 (s, 9H), 0.88 (s, 9H), 0.19 (s, 3H), 0.13 (s, 3H), 0.09 (s, 6H), 0.05 (s, 3H), 0.04 (s, 3H). **^13^C NMR** (101 MHz, CDCl_3_) δ 141.7, 99.3, 80.0, 71.4, 70.7, 61.1, 25.9, 25.7, 25.7, 18.4, 18.1, 18.0, -4.4, -4.6, -4.7, -5.2, -5.3. **HRMS** (ESI) Calculated for C_24_H_55_NBrO_4_Si_3_ [M+NH_4_]^+^: 584.2622 Found: 584.2632.

**2-Bromo-3,4,6-tri-*O*-methyl-D-glucal (3g).** Compound **3g** was prepared as yellow oil (18.2 mg, 68%) from compound **1g** (0.10 mmol, 18.8 mg) after flash column chromatography (petroleum ether: ethyl acetate = 8: 1, v/v) following the general procedure B of the electrochemical bromination. **^1^H NMR** (400 MHz, CDCl_3_) δ 6.65 (d, *J* = 0.9 Hz, 1H), 4.23 – 4.18 (m, 1H), 3.85 – 3.84 (m, 1H), 3.72 – 3.62 (m, 2H), 3.57 (dd, *J* = 10.6, 3.7 Hz, 1H), 3.52 (s, 6H), 3.40 (s, 3H). The ^1^H NMR data are consistent with those reported (Dharuman and Vankar, 2014).

**2-Bromo-3,4,6-tri-*O*-benzyl-D-galactal (3h).** Compound **3h** was prepared as yellow oil (13.9 mg, 56%) from compound **1h** (0.05 mmol, 20.8 mg) after flash column chromatography (petroleum ether: ethyl acetate = 15: 1, v/v) following the general procedure A of the electrochemical bromination. **^1^H NMR** (400 MHz, CDCl_3_) δ 7.41 – 7.27 (m, 15H), 6.58 (d, *J* = 0.9 Hz, 1H), 4.81 – 4.74 (m, 3H), 4.59 (d, *J* = 11.9 Hz, 1H), 4.52 (d, *J* = 11.8 Hz, 1H), 4.42 (d, *J* = 11.9 Hz, 1H), 4.37 – 4.31 (m, 1H), 4.18 – 4.16 (m, 1H), 4.01 (t, *J* = 3.8 Hz, 1H), 3.86 (dd, *J* = 10.9, 7.9 Hz, 1H), 3.73 (dd, *J* = 10.9, 3.8 Hz, 1H). The ^1^H NMR data are consistent with those reported (Dharuman and Vankar, 2014).

**2-Bromo-3,4,6-tri-*O*-(*p*-methoxybenzyl)-D-galactal (3i).** Compound **3i** was prepared as yellow oil (21.4 mg, 73%) from compound **1i** (0.05 mmol, 25.3 mg) after flash column chromatography (petroleum ether: ethyl acetate = 8: 1, v/v) following the general procedure A of the electrochemical bromination. **^1^H NMR** (400 MHz, CDCl_3_) δ 7.33 – 7.28 (m, 2H), 7.22 – 7.18 (m, 4H), 6.89 – 6.83 (m, 6H), 6.55 (d, *J* = 0.9 Hz, 1H), 4.75 – 4.66 (m, 3H), 4.52 (d, *J* = 11.6 Hz, 1H), 4.45 (d, *J* = 11.5 Hz, 1H), 4.33 (d, *J* = 11.5 Hz, 1H), 4.30 – 4.23 (m, 1H), 4.13 – 4.11 (m, 1H), 3.95 (t, *J* = 3.8 Hz, 1H), 3.82 (s, 3H), 3.80 (s, 6H), 3.78 – 3.74 (m, 1H), 3.65 (dd, *J* = 10.9, 3.9 Hz, 1H). **^13^C NMR** (101 MHz, CDCl_3_) δ 159.4, 159.3, 159.2, 143.3, 130.3, 130.1, 129.9, 129.6, 129.6, 129.5, 113.8, 113.8, 113.7, 98.8, 75.9, 74.1, 73.7, 73.1, 72.9, 72.6, 67.4, 55.3, 55.3. **HRMS** (ESI) Calculated for C_30_H_37_NBrO_7_ [M+NH_4_]^+^: 602.1753 Found: 602.1755.

**2-Bromo-3,4,6-tri-*O*-(*tert*-butyldimethylsilyl)-D-galactal (3j).** Compound **3j** was prepared as yellow oil (20.2 mg, 71%) from compound **1j** (0.05 mmol, 24.5 mg) after flash column chromatography (petroleum ether: ethyl acetate = 15: 1, v/v) following the general procedure A of the electrochemical bromination. **^1^H NMR** (400 MHz, CDCl_3_) δ 6.48 (s, 1H), 4.21 – 4.04 (m, 4H), 3.88 (d, *J* = 10.7 Hz, 1H), 0.92 (s, 18H), 0.89 (s, 9H), 0.19 (s, 3H), 0.13 (s, 6H), 0.11 (s, 3H), 0.05 (s, 6H). **^13^C NMR** (101 MHz, CDCl_3_) δ 141.9, 98.6, 79.9, 71.2, 70.2, 60.5, 26.1, 26.0, 18.5, -4.2, -4.3, -4.9, -5.1, -5.3. **HRMS** (ESI) Calculated for C_24_H_52_BrO_4_Si_3_ [M+H]^+^: 567.2351 Found: 567.2361.

**2-Bromo-3,4,6-tri-*O*-acetyl-D-galactal (3k).** Compound **3k** was prepared as yellow oil (11.0 mg, 39%) from compound **1k** (0.05 mmol, 13.6 mg) after flash column chromatography (petroleum ether: ethyl acetate = 6: 1, v/v) following the general procedure C of the electrochemical bromination. **^1^H NMR** (400 MHz, CDCl_3_) δ 6.74 (d, *J* = 1.4 Hz, 1H), 5.70 – 5.69 (m, 1H), 5.50 (dd, *J* = 4.7, 2.3 Hz, 1H), 4.42 – 4.36 (m, 1H), 4.30 (dd, *J* = 11.7, 7.5 Hz, 1H), 4.21 (dd, *J* = 11.7, 5.0 Hz, 1H), 2.13 (s, 3H), 2.10 (s, 3H), 2.09 (s, 3H). The ^1^H NMR data are consistent with those reported (Leibeling et al., 2010).

**2-Bromo-3,4,6-tri-*O*-benzoyl-D-galactal (3l).** Compound **3l** was prepared as yellow oil (15.0 mg, 56%) from compound **1l** (0.05 mmol, 22.9 mg) after flash column chromatography (petroleum ether: ethyl acetate = 8: 1, v/v) following the general procedure C of the electrochemical bromination. **^1^H NMR** (400 MHz, CDCl_3_) δ 8.05 – 8.01 (m, 2H), 7.95 – 7.93 (m, 4H), 7.60 – 7.51 (m, 3H), 7.46 – 7.33 (m, 6H), 6.92 (d, *J* = 1.3 Hz, 1H), 6.15 – 6.10 (m, 1H), 5.98 (dd, *J* = 4.8, 2.4 Hz, 1H), 4.86 – 4.73 (m, 2H), 4.54 (dd, *J* = 11.2, 3.9 Hz, 1H). **^13^C NMR** (101 MHz, CDCl_3_) δ 166.2, 165.5, 165.2, 145.4, 133.6, 133.4, 133.3, 130.0, 129.9, 129.8, 129.4, 129.0, 128.9, 128.5, 128.5, 128.4, 96.4, 73.6, 66.6, 65.7, 62.0. **HRMS** (ESI) Calculated for C_27_H_25_NBrO_7_ [M+NH_4_]^+^: 554.0814 Found: 554.0823.

**2-Bromo-3,4-di-*O*-acetyl-L-rhamnal (3m).** Compound **3m** was prepared as yellow oil (13.0 mg, 89%) from compound **1m** (0.05 mmol, 10.7 mg) after flash column chromatography (petroleum ether: ethyl acetate = 10: 1, v/v) following the general procedure C of the electrochemical bromination. **^1^H NMR** (400 MHz, CDCl_3_) δ 6.74 (d, *J* = 1.1 Hz, 1H), 5.52 – 5.51 (m, 1H), 5.03 (dd, *J* = 6.3, 4.7 Hz, 1H), 4.37 – 4.17 (m, 1H), 2.12 (s, 3H), 2.10 (s, 3H), 1.34 (d, *J* = 6.7 Hz, 3H). The ^1^H NMR data are consistent with those reported (Fraser-Reid et al., 2000).

**2-Bromo-3,4-di-*O*-benzyl-L-rhamnal (3n).** Compound **3n** was prepared as yellow oil (16.4 mg, 82%) from compound **1n** (0.05 mmol, 15.5 mg) after flash column chromatography (petroleum ether: ethyl acetate = 12: 1, v/v) following the general procedure A of the electrochemical bromination. **^1^H NMR** (400 MHz, CDCl_3_) δ 7.42 – 7.27 (m, 10H), 6.65 (d, *J* = 1.0 Hz, 1H), 4.79 – 4.72 (m, 2H), 4.66 – 4.60 (m, 2H), 4.23 – 4.11 (m, 2H), 3.62 (dd, *J* = 6.7, 5.0 Hz, 1H), 1.36 (d, *J* = 6.6 Hz, 3H). **^13^C NMR** (101 MHz, CDCl_3_) δ 144.4, 137.9, 137.6, 128.5, 128.4, 128.0, 128.0, 127.9, 127.9, 99.0, 78.7, 78.2, 74.0, 73.2, 72.5, 16.7. **HRMS** (ESI) Calculated for C_20_H_25_NBrO_3_ [M+ NH_4_]^+^: 406.1018 Found: 406.1017.

**2-Bromo-3,4-di-*O*-(*p*-methoxybenzyl)-L-rhamnal (3o).** Compound **3o** was prepared as yellow oil (14.8 mg, 66%) from compound **1o** (0.05 mmol, 18.5 mg) after flash column chromatography (petroleum ether: ethyl acetate = 10: 1, v/v) following the general procedure A of the electrochemical bromination. **^1^H NMR** (400 MHz, CDCl_3_) δ 7.34 – 7.29 (m, 2H), 7.25 – 7.20 (m, 2H), 6.91 – 6.85 (m, 4H), 6.62 (d, *J* = 1.0 Hz, 1H), 4.69 (t, *J* = 10.7 Hz, 2H), 4.60 – 4.53 (m, 2H), 4.17 – 4.08 (m, 2H), 3.81 (s, 6H), 3.57 (dd, *J* = 6.9, 5.0 Hz, 1H), 1.34 (d, *J* = 6.6 Hz, 3H). **^13^C NMR** (101 MHz, CDCl_3_) δ 159.4, 159.4, 144.3, 130.1, 129.8, 129.7, 129.6, 113.9, 113.8, 99.3, 78.4, 78.1, 74.1, 73.0, 72.2, 55.3, 16.7. **HRMS** (ESI) Calculated for C_29_H_35_NBrO_7_ [M+NH_4_]^+^: 466.1229 Found: 466.1226.

**2-Bromo-3,4-di-*O*-benzyl-L-arabinal (3p).** Compound **3p** was prepared as yellow oil (11.4 mg, 61%) from compound **1p** (0.05 mmol, 14.8 mg) after flash column chromatography (petroleum ether: ethyl acetate = 12: 1, v/v) following the general procedure A of the electrochemical bromination. **^1^H NMR** (400 MHz, CDCl_3_) δ 7.46 – 7.26 (m, 10H), 6.62 (s, 1H), 4.94 (d, *J* = 11.4 Hz, 1H), 4.83 (d, *J* = 11.4 Hz, 1H), 4.66 (d, *J* = 12.0 Hz, 1H), 4.59 (d, *J* = 12.0 Hz, 1H), 4.20 – 4.16 (m, 1H), 4.05 – 3.97 (m, 2H), 3.88 – 3.83 (m, 1H). **^13^C NMR** (101 MHz, CDCl_3_) δ 145.5, 138.3, 137.6, 128.6, 128.3, 128.2, 128.0, 127.7, 127.6, 96.5, 74.4, 74.1, 74.0, 71.6, 62.6. **HRMS** (ESI) Calculated for C_19_H_23_NBrO_3_ [M+NH_4_]^+^: 392.0861 Found: 392.0856.

**2-Bromo-3,4-di-*O*-(*p*-methoxybenzyl)-L-arabinal (3q).** Compound **3q** was prepared as yellow oil (16.0 mg, 74%) from compound **1q** (0.05 mmol, 17.8 mg) after flash column chromatography (petroleum ether: ethyl acetate = 8: 1, v/v) following the general procedure A of the electrochemical bromination. **^1^H NMR** (400 MHz, CDCl_3_) δ 7.38 – 7.33 (m, 2H), 7.29 – 7.23 (m, 2H), 6.92 – 6.83 (m, 4H), 6.60 (s, 1H), 4.86 (d, *J* = 11.1 Hz, 1H), 4.74 (d, *J* = 11.1 Hz, 1H), 4.58 (d, *J* = 11.6 Hz, 1H), 4.52 (d, *J* = 11.6 Hz, 1H), 4.17 – 4.11 (m, 1H), 4.01 – 3.92 (m, 2H), 3.84 – 3.79 (m, 7H). **^13^C NMR** (101 MHz, CDCl_3_) δ 159.5, 159.3, 145.4, 130.5, 129.9, 129.7, 129.3, 113.9, 113.7, 96.7, 74.1, 73.7, 73.6, 71.3, 62.7, 55.3. **HRMS** (ESI) Calculated for C_21_H_27_NBrO_5_ [M+NH_4_]^+^: 452.1073 Found: 452.1069.

**2-Bromo-3,4-di-*O*-benzyl-D-xylal (3r).** Compound **3r** was prepared as yellow oil (18.0 mg, 96%) from compound **1r** (0.05 mmol, 14.8 mg) after flash column chromatography (petroleum ether: ethyl acetate = 10: 1, v/v) following the general procedure A of the electrochemical bromination. **^1^H NMR** (400 MHz, CDCl_3_) δ 7.40 – 7.28 (m, 10H), 6.79 (s, 1H), 4.68 (d, *J* = 11.7 Hz, 1H), 4.62 (d, *J* = 11.7 Hz, 1H), 4.56 (s, 2H), 4.21 – 4.17 (m, 1H), 3.92 – 3.86 (m, 2H), 3.69 – 3.67 (m, 1H). **^13^C NMR** (151 MHz, CDCl_3_) δ 145.6, 137.7, 137.4, 128.6, 128.5, 128.1, 128.1, 128.0, 127.8, 97.6, 74.2, 73.0, 72.2, 71.2, 63.5. **HRMS** (ESI) Calculated for C_19_H_23_NBrO_3_ [M+ NH_4_]^+^: 392.0861 Found: 392.0862.

**2-Bromo-3,4-di-*O*-(*p*-methoxybenzyl)-D-xylal (3s).** Compound **3s** was prepared as yellow oil (21.0 mg, 97%) from compound **1s** (0.05 mmol, 17.8 mg) after flash column chromatography (petroleum ether: ethyl acetate = 10: 1, v/v) following the general procedure A of the electrochemical bromination. **^1^H NMR** (400 MHz, CDCl_3_) δ 7.27 – 7.21 (m, 4H), 6.91 – 6.85 (m, 4H), 6.77 (s, 1H), 4.60 (d, *J* = 11.5 Hz, 1H), 4.56 (d, *J* = 11.5 Hz, 1H), 4.49 (s, 2H), 4.16 – 4.12 (m, 1H), 3.92 – 3.83 (m, 2H), 3.81 (s, 3H), 3.81 (s, 3H), 3.65 – 3.63 (m, 1H). **^13^C NMR** (101 MHz, CDCl_3_) δ 159.5, 159.4, 145.5, 129.8, 129.7, 129.5, 129.4, 114.0, 113.8, 97.8, 73.8, 72.7, 71.9, 70.8, 63.6, 55.3. **HRMS** (ESI) Calculated for C_21_H_27_NBrO_5_ [M+NH_4_]^+^: 452.1073 Found: 452.1071.

**2’-Bromo-2,3,3’,4,6,6’-hexa-*O*-benzyl-D-lactal (3t).** Compound **3t** was prepared as yellow oil (33.6 mg, 72%) from compound **1t** (0.05 mmol, 42.5 mg) after flash column chromatography (petroleum ether: ethyl acetate = 10: 1, v/v) following the general procedure A of the electrochemical bromination. **^1^H NMR** (400 MHz, CDCl_3_) δ 7.35 – 7.26 (m, 30H), 6.68 (s, 1H), 4.94 (d, *J* = 11.7 Hz, 1H), 4.84 (d, *J* = 10.7 Hz, 1H), 4.78 – 4.69 (m, 3H), 4.67 (s, 2H), 4.61 (d, *J* = 11.7 Hz, 1H), 4.50 – 4.40 (m, 4H), 4.39 – 4.30 (m, 2H), 4.24 (t, *J* = 3.3 Hz, 1H), 4.16 (s, 1H), 3.86 (d, *J* = 2.9 Hz, 1H), 3.81 – 3.76 (m, 2H), 3.61 (dd, *J* = 10.5, 4.8 Hz, 1H), 3.53 – 3.43 (m, 4H). **^13^C NMR** (101 MHz, CDCl_3_) δ 143.4, 138.5, 138.5, 138.4, 138.1, 137.9, 137.8, 128.4, 128.4, 128.4, 128.3, 128.2, 128.2, 127.9, 127.9, 127.8, 127.7, 127.7, 127.6, 127.6, 127.5, 127.5, 103.2, 98.2, 82.1, 79.1, 76.2, 75.3, 75.2, 74.6, 73.7, 73.6, 73.5, 73.2, 73.1, 72.3, 68.8, 67.3. **HRMS** (ESI) Calculated for C_54_H_59_NBrO_9_ [M+NH_4_]^+^: 944.3373 Found: 944.3387.

1. **The synthesis of compounds 5a-c**

**The synthesis of compound 5a.** A 25 mL two-neck flask was charged with compound **3h** (0.10 mmol, 50 mg), phenylacetylene **4a** (0.20 mmol, 22.2 μL), Pd(PPh_3_)_4_Cl_2_ (0.005 mmol, 3.5 mg), CuI (0.01 mmol, 1.92 mg) and Et_3_N (2.0 mL). The reaction mixture was sealed, degassed and filled with argon. After stirring at 80 ^o^C in an oil bath for 3.5 h, the reaction mixture was filtered through a pad of celite and concentrated under reduced pressure. Compound **5a** was obtained as yellow oil (41.8 mg, 80%) after flash column chromatography (petroleum ether: ethyl acetate = 60: 1, v/v). **^1^H NMR** (400 MHz, CDCl_3_) δ 7.46 – 7.26 (m, 20H), 6.85 (d, *J* = 0.9 Hz, 1H), 4.94 (d, *J* = 12.1 Hz, 1H), 4.87 – 4.80 (m, 2H), 4.62 (d, *J* = 12.0 Hz, 1H), 4.53 (d, *J* = 11.9 Hz, 1H), 4.44 (d, *J* = 11.9 Hz, 1H), 4.36 – 4.30 (m, 1H), 4.28 – 4.26 (m, 1H), 3.95 (t, *J* = 3.6 Hz, 1H), 3.87 (dd, *J* = 10.7, 7.6 Hz, 1H), 3.75 (dd, *J* = 10.8, 4.1 Hz, 1H). **^13^C NMR** (101 MHz, CDCl_3_) δ 150.1, 138.5, 138.0, 137.9, 131.1, 128.4, 128.3, 128.2, 128.0, 127.9, 127.8, 127.8, 127.7, 127.6, 127.6, 123.9, 98.3, 89.0, 86.7, 76.4, 73.5, 73.0, 72.9, 71.8, 71.6, 68.3. **HRMS** (ESI) Calculated for C_35_H_36_NO_4_ [M+NH_4_]^+^: 534.2644 Found: 534.2640.

**The synthesis of compound 5b.** A 25 mL two-neck flask was charged with compound **3h** (0.10 mmol, 50 mg), potassium phenyltrifluoroborate **4b** (0.11 mmol, 20.4 mg), Pd(PPh_3_)_4_ (0.002 mmol, 2.3 mg), K_2_CO_3_ (0.30 mmol, 98.7 mg), toluene (0.8 mL) and H_2_O (0.2 mL). The reaction mixture was sealed, degassed and filled with argon. After stirring at 90 ^o^C in an oil bath for 3 h, the reaction mixture was filtered through a pad of celite and concentrated under reduced pressure. Compound **5b** was obtained as colorless oil (39.4 mg, 79%) after flash column chromatography (petroleum ether: ethyl acetate = 60: 1, v/v). **^1^H NMR** (400 MHz, CDCl_3_) δ 7.40 – 7.08 (m, 20H), 6.64 (s, 1H), 4.80 – 4.76 (m, 2H), 4.70 (d, *J* = 11.9 Hz, 1H), 4.63 – 4.46 (m, 5H), 4.14 – 4.04 (m, 2H), 3.89 (dd, *J* = 11.2, 2.7 Hz, 1H). **^13^C NMR** (101 MHz, CDCl_3_) δ 142.0, 138.4, 138.3, 138.0, 137.4, 128.5, 128.4, 128.4, 128.2, 127.9, 127.9, 127.8, 127.7, 127.6, 127.4, 126.3, 126.0, 114.0, 75.3, 74.7, 73.6, 73.4, 72.4, 71.1, 68.1. **HRMS** (ESI) Calculated for C_33_H_36_NO_4_ [M+NH_4_]^+^: 510.2644 Found: 510.2639.

**The synthesis of compound 5c.** A 25 mL oven-dried two-neck flask was charged with compound **3h** (0.10 mmol, 50 mg), compound **4c** (0.07 mmol, 31.3 mg), activated 4 Å molecular sieves (100 mg) and dry CH_2_Cl_2_ (2.0 mL) under an argon atmosphere. After stirring for 10 min, BF_3_·Et_2_O (0.17 mmol, 20.7 μL) was added at 0 °C, and then the mixture was stirred at room temperature for 2 h. After completion, the reaction was quenched with triethylamine (0.1 mL). The reaction mixture was filtered through a pad of celite and concentrated under reduced pressure. Compound **5c** was obtained as colorless oil (40.7 mg, 72%) after flash column chromatography (petroleum ether: ethyl acetate = 8: 1, v/v). Compound **5cα: ^1^H NMR** (400 MHz, CDCl_3_) δ 7.37 – 7.27 (m, 20H), 6.36 (d, *J* = 5.6 Hz, 1H), 5.19 (s, 1H), 4.96 (d, *J* = 10.9 Hz, 1H), 4.88 – 4.75 (m, 3H), 4.69 – 4.47 (m, 7H), 4.29 – 4.25 (m, 1H), 3.99 (t, *J* = 9.3 Hz, 1H), 3.87 (d, *J* = 3.1 Hz, 2H), 3.81 – 3.73 (m, 3H), 3.70 – 3.60 (m, 2H), 3.52 (dd, *J* = 9.7, 3.5 Hz, 1H), 3.36 (s, 3H). **^13^C NMR** (101 MHz, CDCl_3_) δ 138.8, 138.3, 138.2, 138.1, 137.9, 128.4, 128.4, 128.4, 128.4, 128.3, 128.1, 128.0, 127.9, 127.8, 127.8, 127.7, 127.6, 127.6, 125.3, 124.8, 98.0, 97.6, 81.9, 79.9, 77.8, 75.8, 75.1, 73.4, 71.3, 70.8, 69.8, 68.8, 68.8, 66.5, 55.2. **HRMS** (ESI) Calculated for C_54_H_59_NBrO_9_ [M+NH_4_]^+^: 868.3060 Found: 868.3066.

1. **References**

Dharuman, S., and Vankar, Y. D. (2014). *N*-Halosuccinimide/AgNO_3_-Efficient Reagent Systems for One-Step Synthesis of 2-Haloglycals from Glycals: Application in the Synthesis of 2C-Branched Sugars via Heck Coupling Reactions. *Org. Lett.* 16, 1172-1175. doi:10.1021/ol500039s

Fraser-Reid, B., Chen, X. T., Haag, D., Henry, K. J., and McPhail, A. T. (2000). IMDA/aldol strategy for transforming carbohydrates into functionalized trans-decalins. *Chirality*. 12, 488-495. doi:10.1002/(sici)1520-636x(2000)12:5/6<488::aid-chir34>3.0.co;2-w

Leibeling, M., Koester, D. C., Pawliczek, M., Schild, S. C., and Werz, D. B. (2010). Domino access to highly substituted chromans and isochromans from carbohydrates. *Nat. Chem. Biol.* 6, 199-201. doi:10.1038/nchembio.302

**5. ^1^H and ^13^C NMR Spectra**


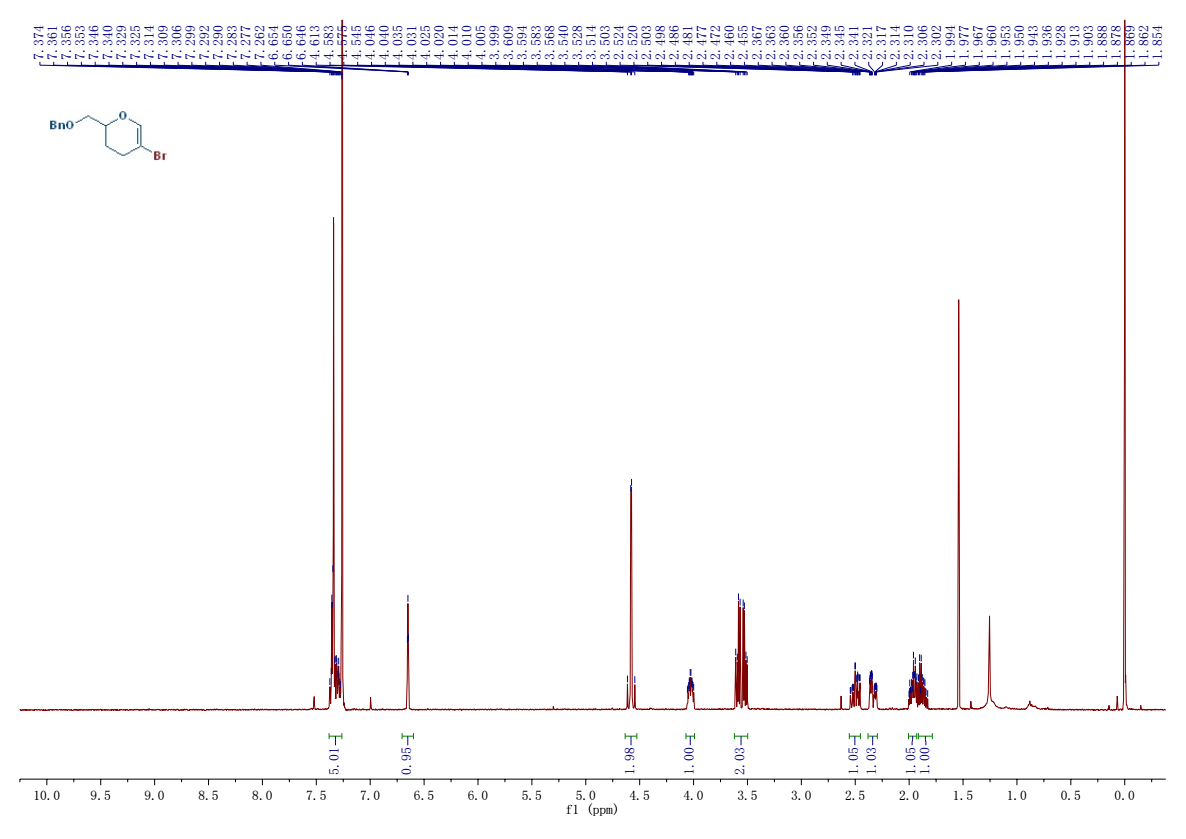


**Supplementary Figure 1. ^1^H NMR spectrum of 3a (400 MHz, CDCl_3_)**


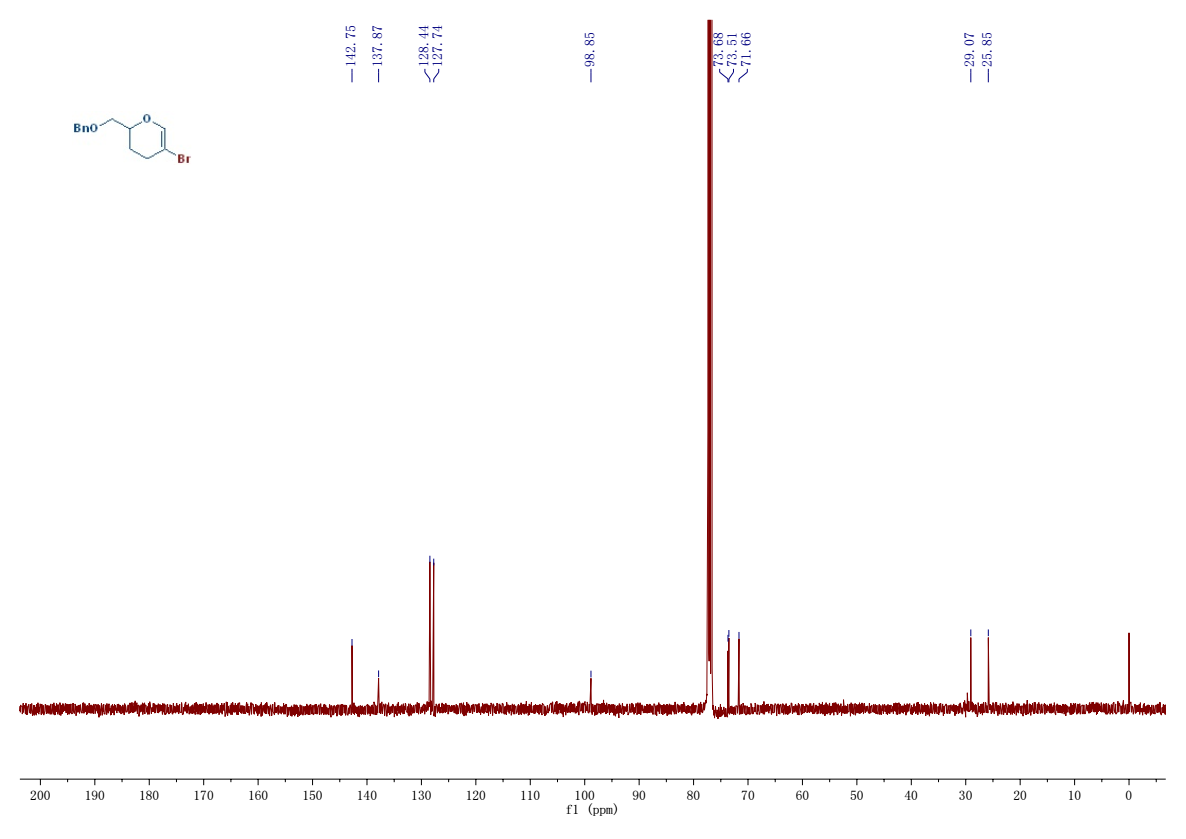


**Supplementary Figure 2. ^13^C NMR spectrum of 3a (101 MHz, CDCl_3_)**


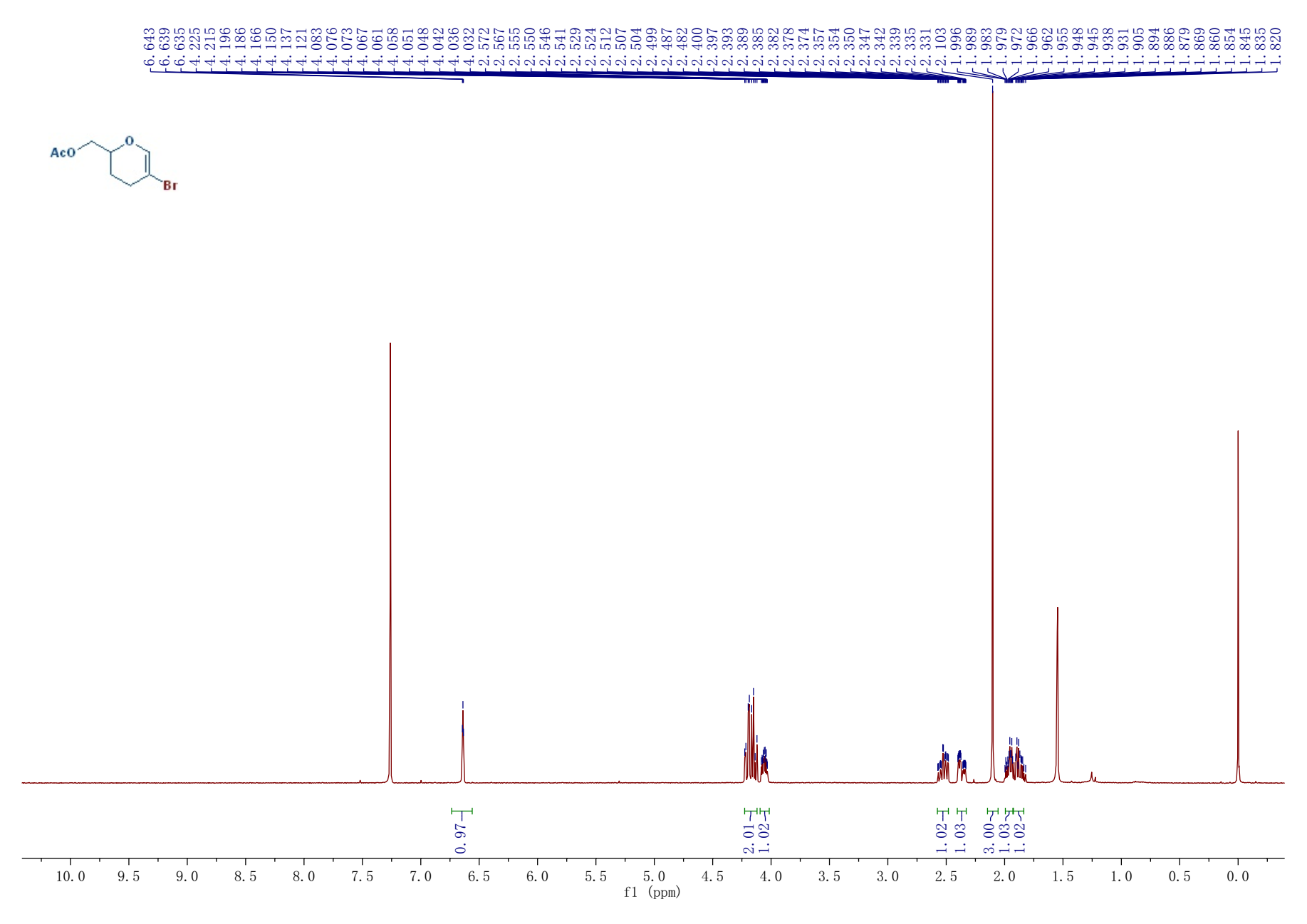


**Supplementary Figure 3. ^1^H NMR spectrum of 3b (400 MHz, CDCl_3_)**


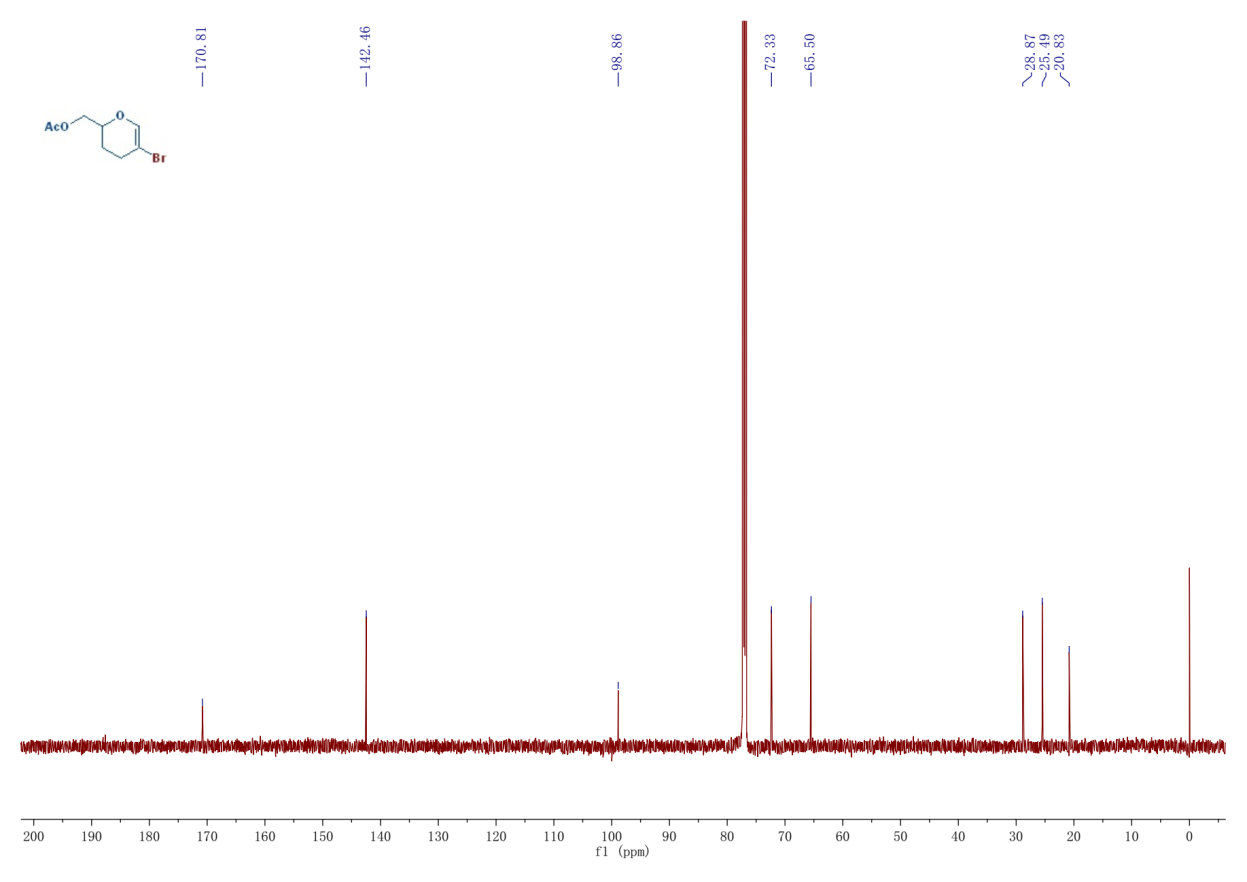


**Supplementary Figure 4. ^13^C NMR spectrum of 3b (151 MHz, CDCl_3_)**


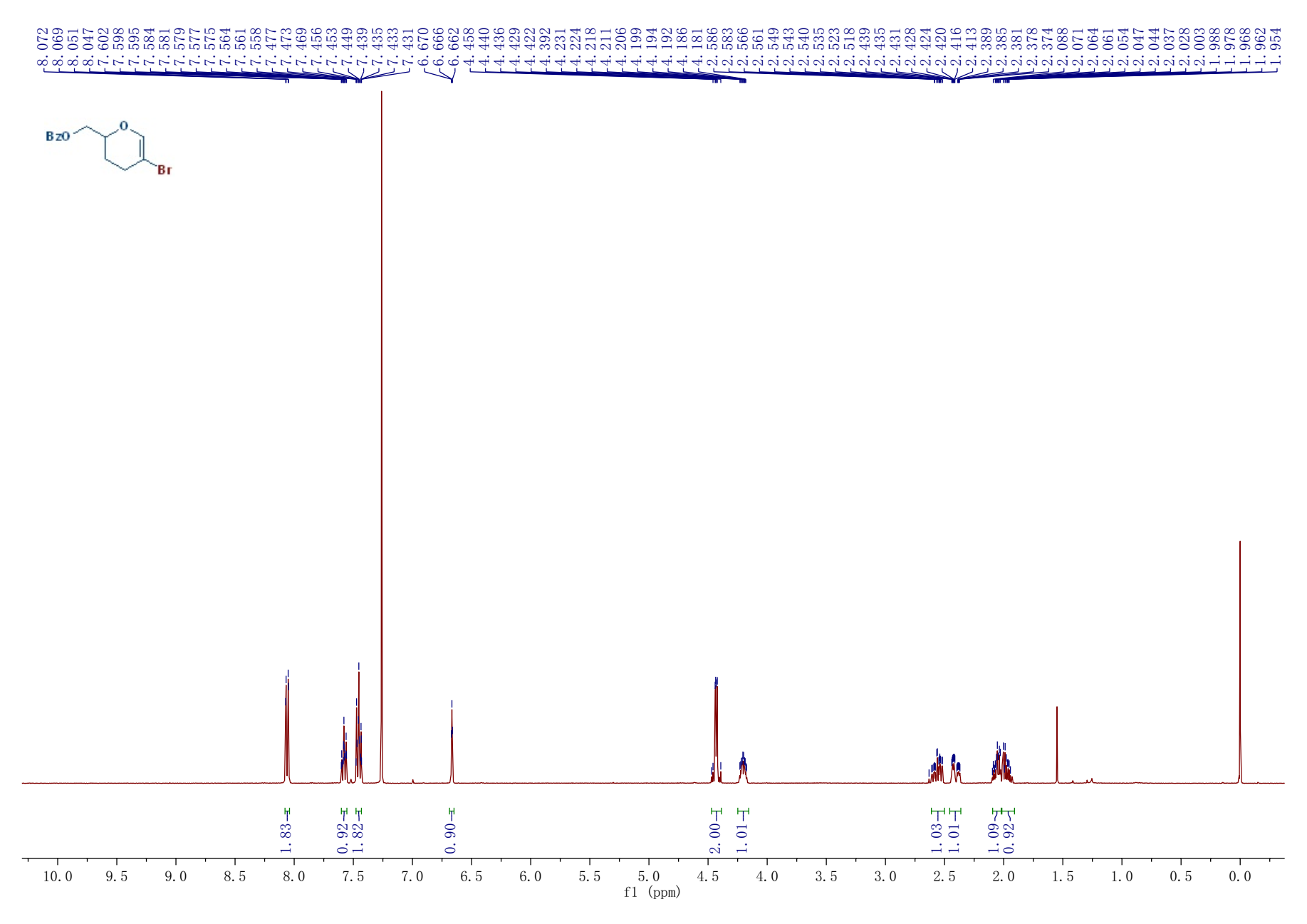


**Supplementary Figure 5. ^1^H NMR spectrum of 3c (400 MHz, CDCl_3_)**


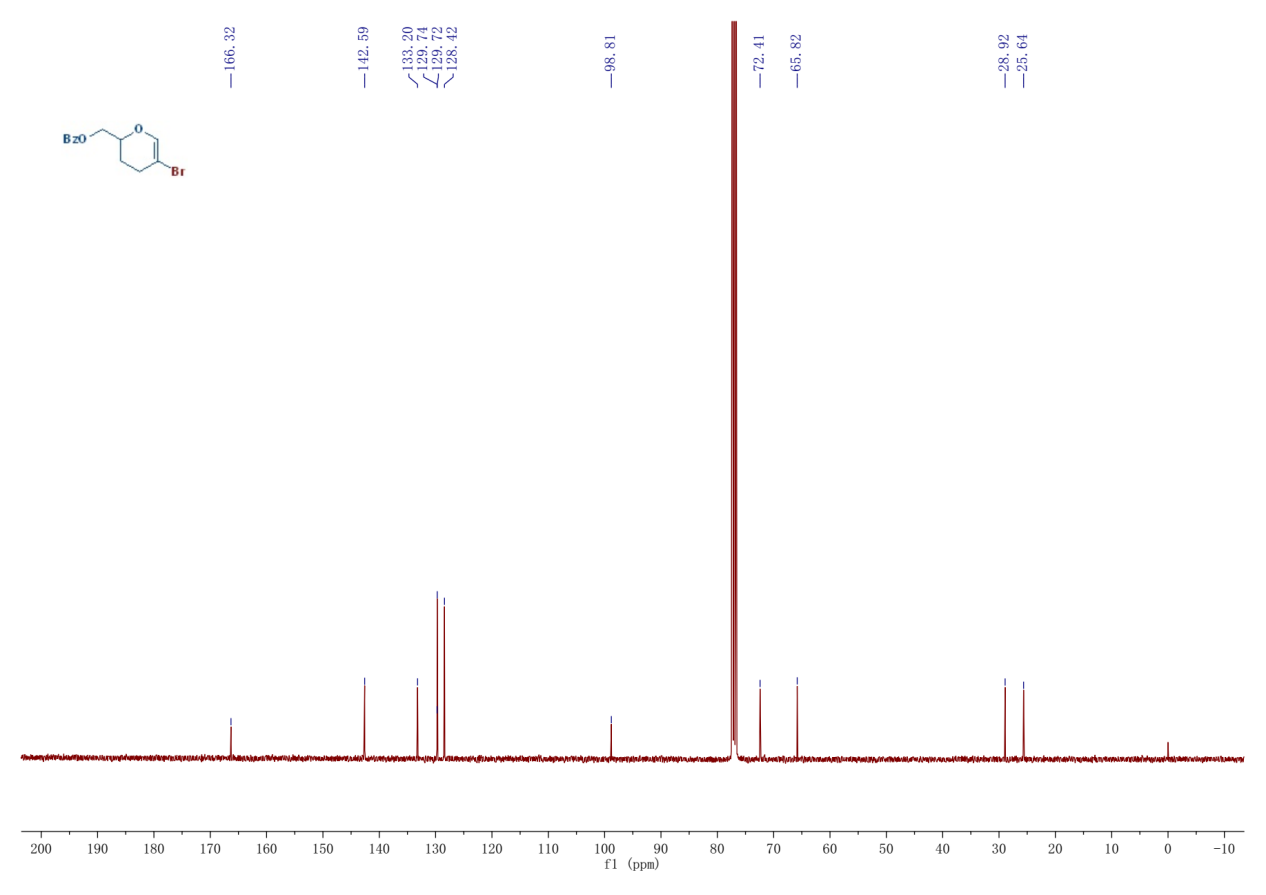


**Supplementary Figure 6. ^13^C NMR spectrum of 3c (101 MHz, CDCl_3_)**


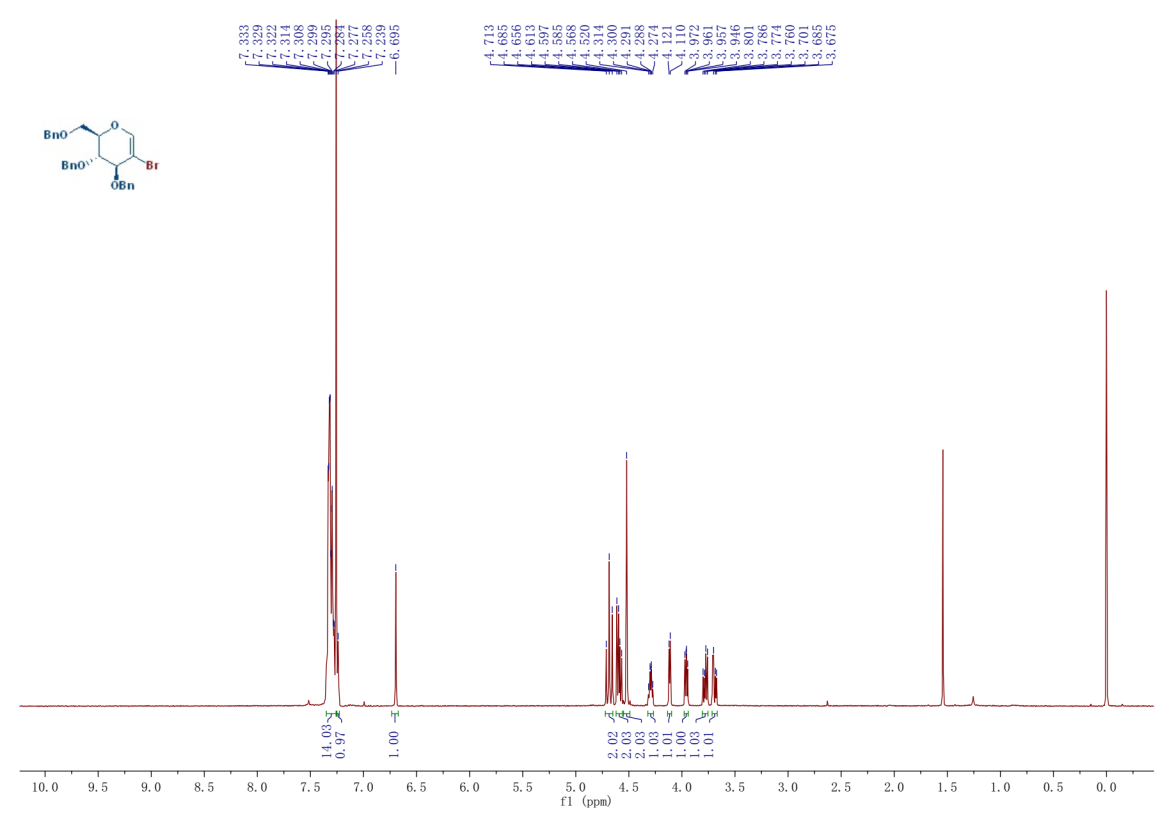


**Supplementary Figure 7. ^1^H NMR spectrum of 3d (400 MHz, CDCl_3_)**


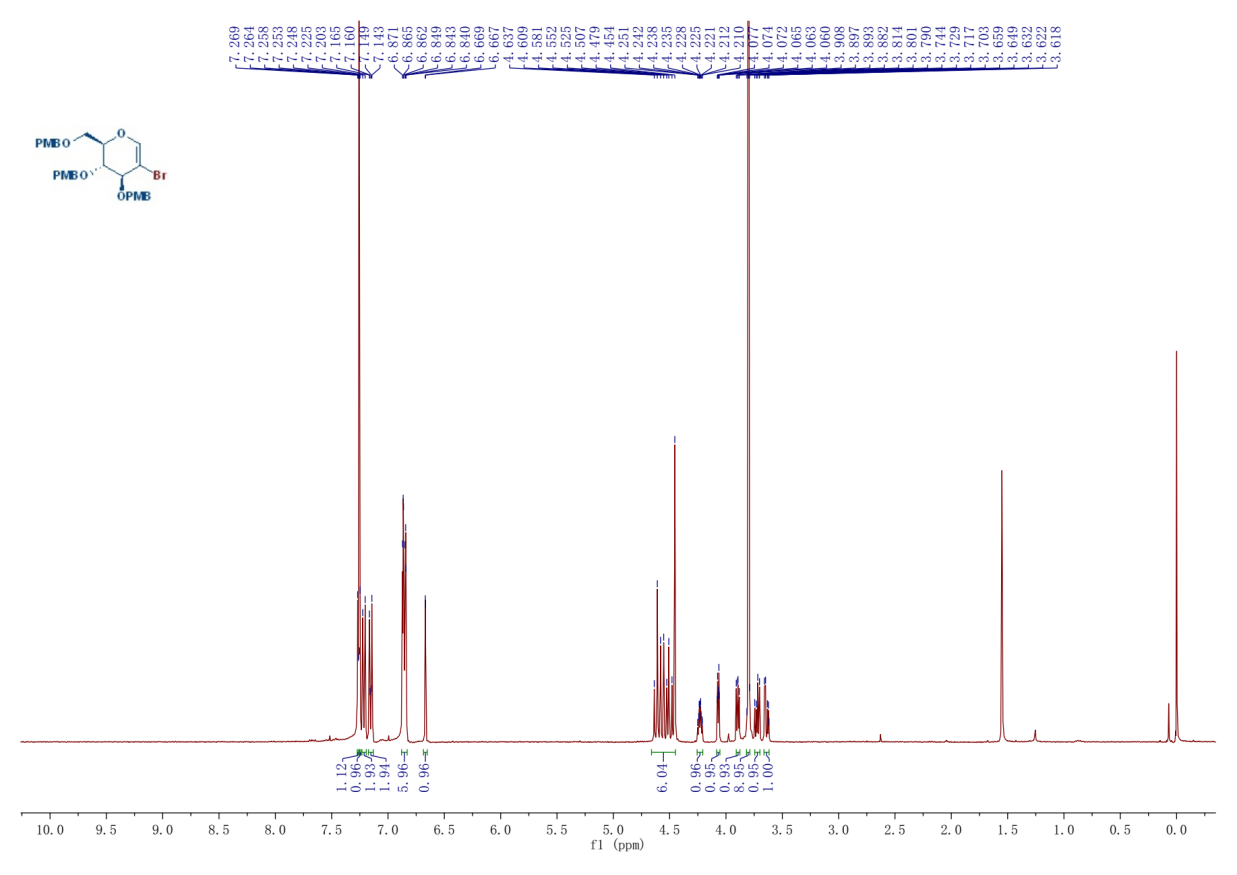


**Supplementary Figure 8. ^1^H NMR spectrum of 3e (400 MHz, CDCl_3_)**


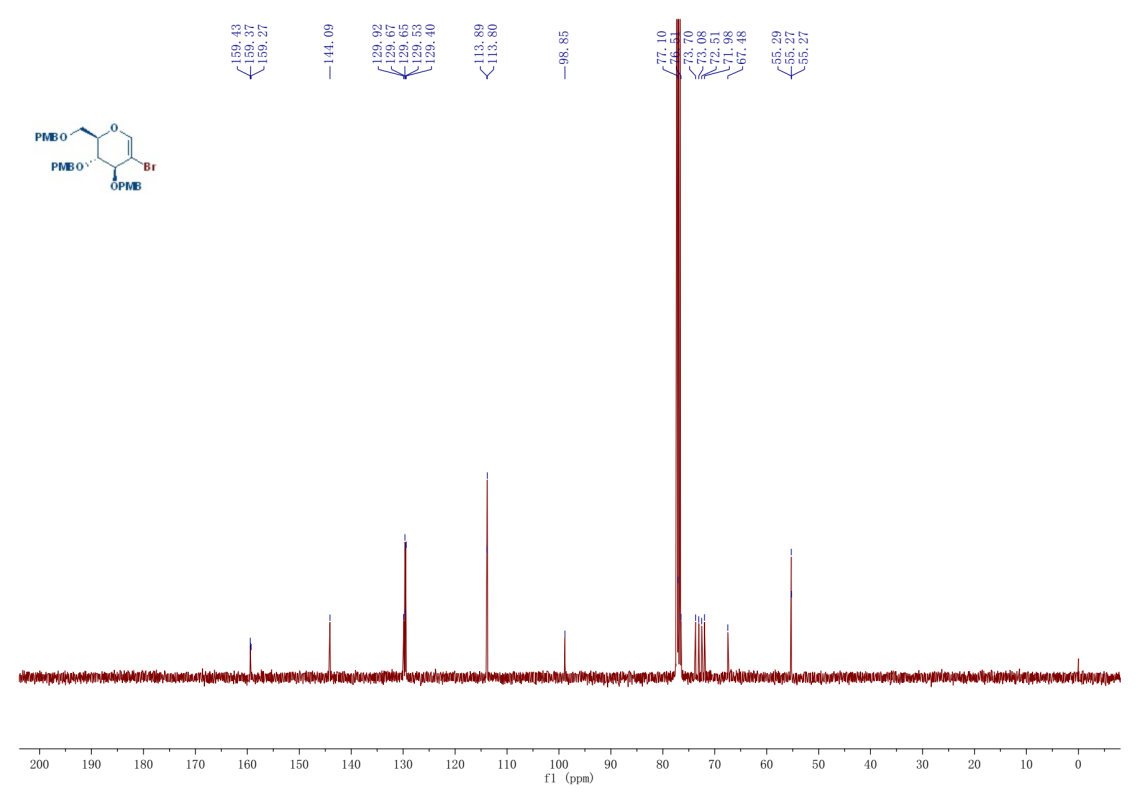


**Supplementary Figure 9. ^13^C NMR spectrum of 3e (101 MHz, CDCl_3_)**


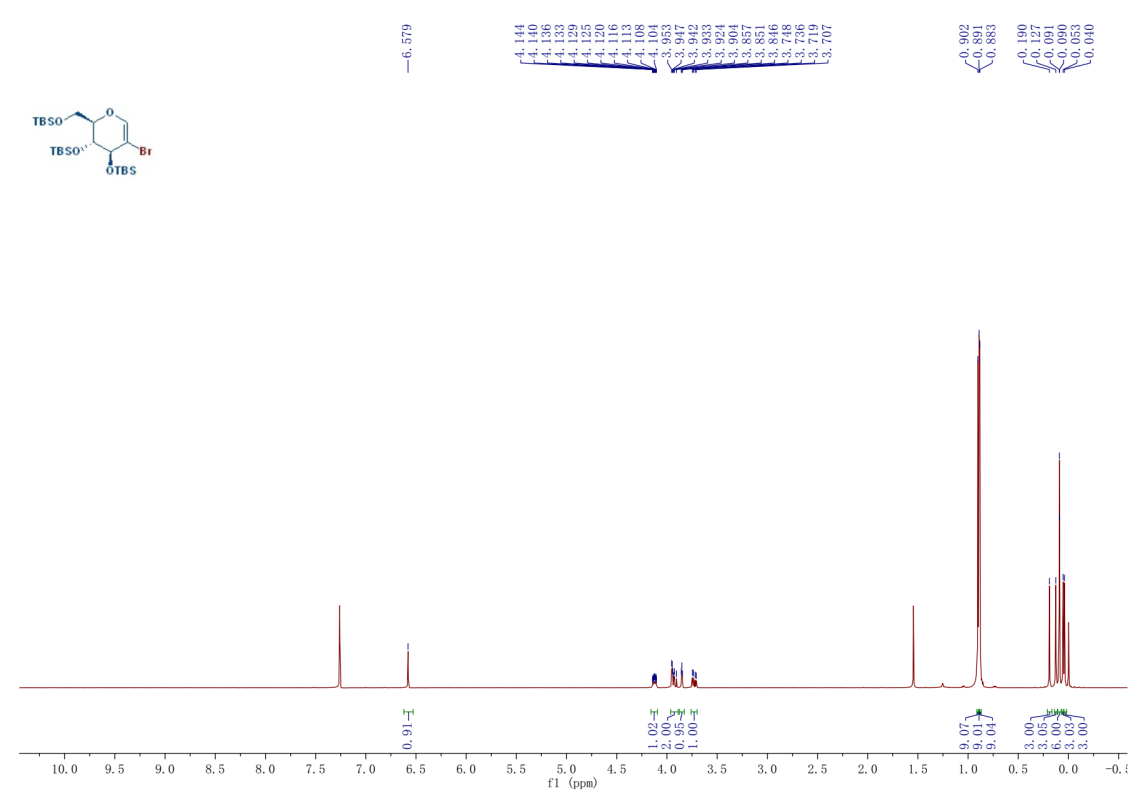


**Supplementary Figure 10. ^1^H NMR spectrum of 3f (400 MHz, CDCl_3_)**


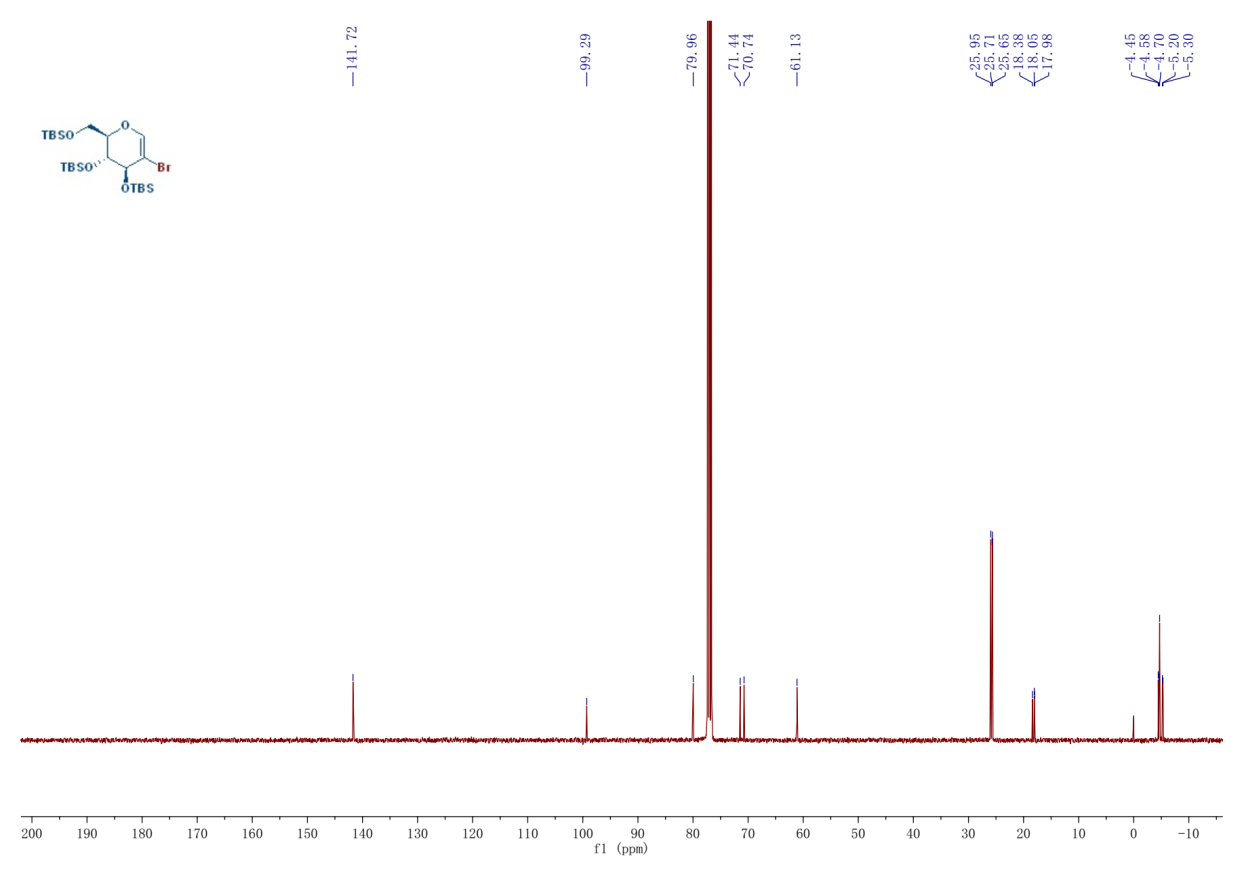


**Supplementary Figure 11. ^13^C NMR spectrum of 3f (101 MHz, CDCl_3_)**


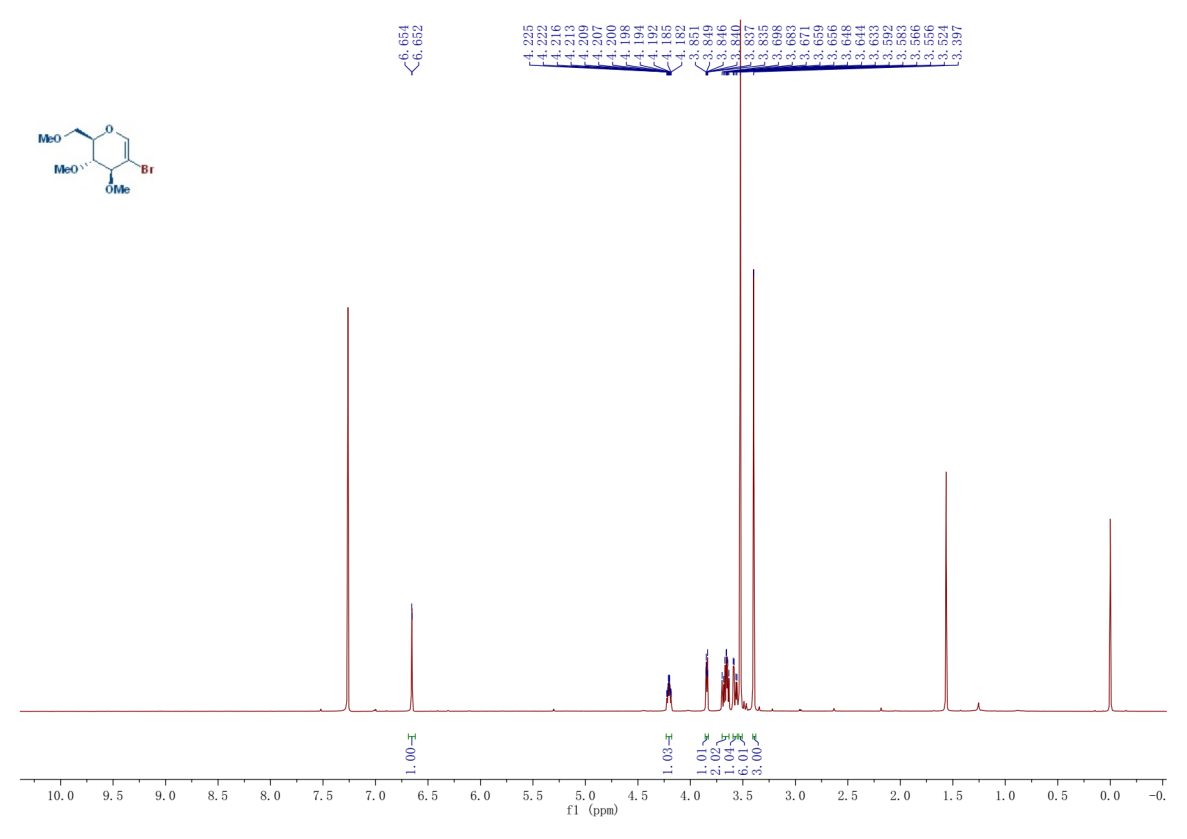


**Supplementary Figure 12. ^1^H NMR spectrum of 3g (400 MHz, CDCl_3_)**


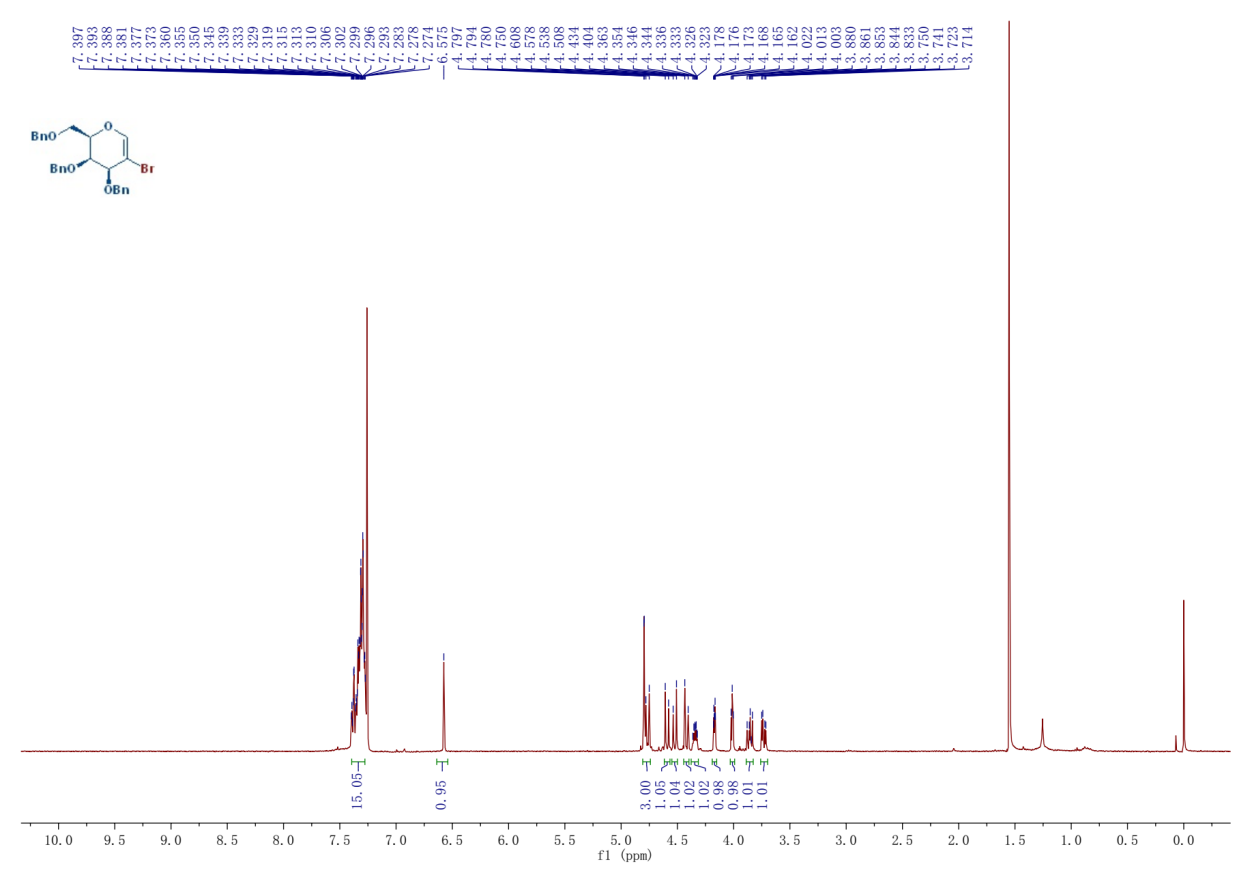


**Supplementary Figure 13. ^1^H NMR spectrum of 3h (400 MHz, CDCl_3_)**


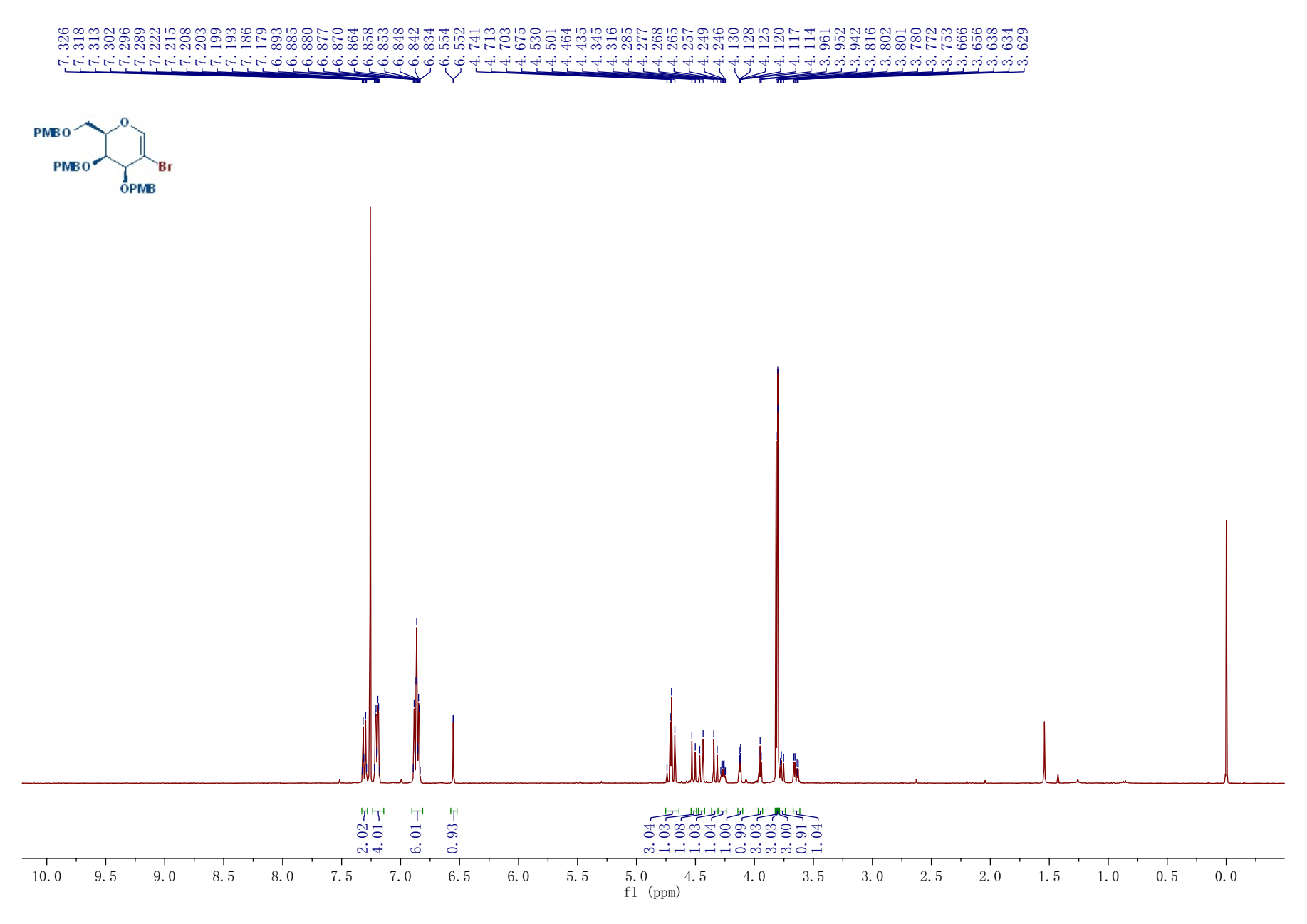


**Supplementary Figure 14. ^1^H NMR spectrum of 3i (400 MHz, CDCl_3_)**


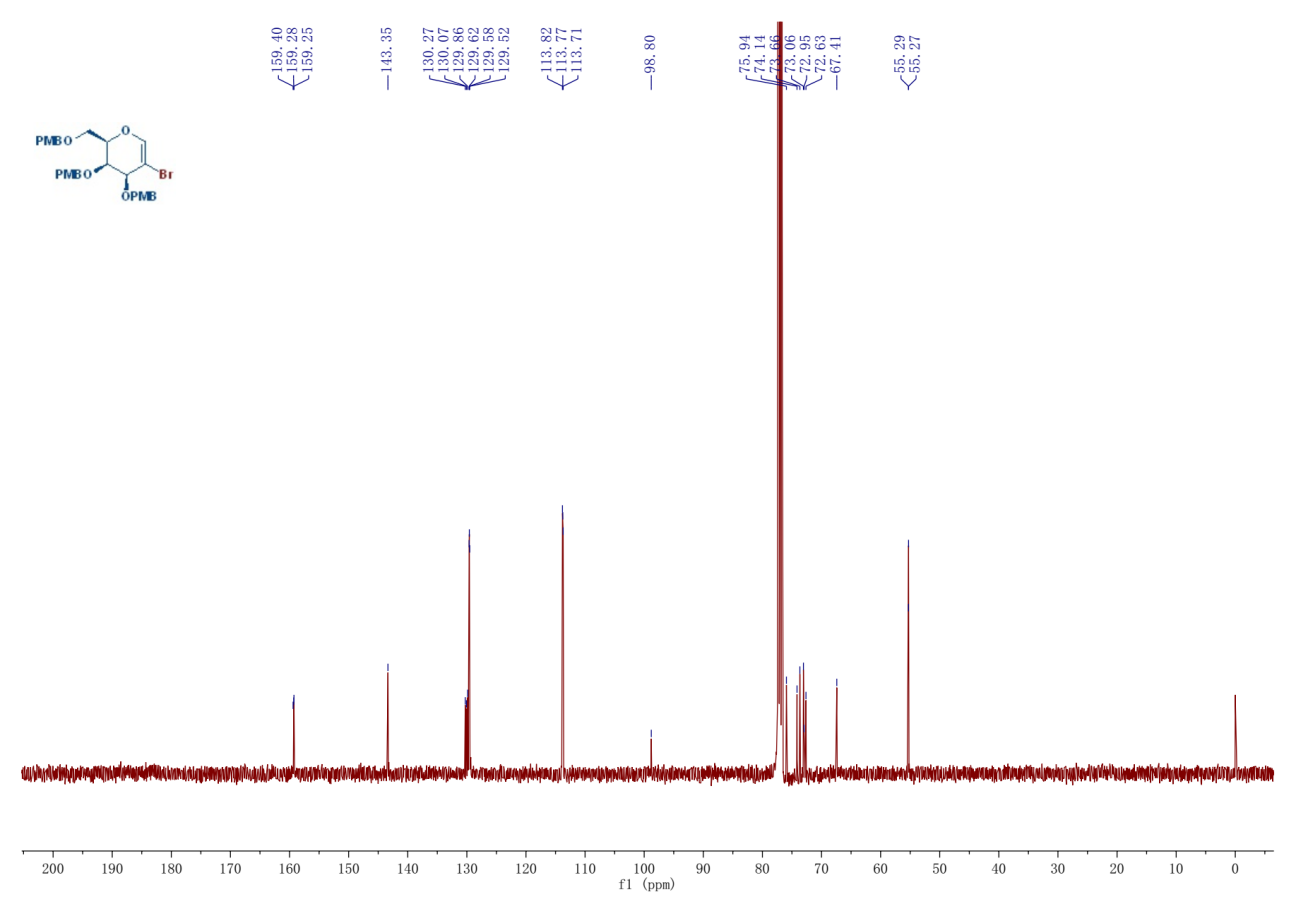


**Supplementary Figure 15. ^13^C NMR spectrum of 3i (101 MHz, CDCl_3_)**


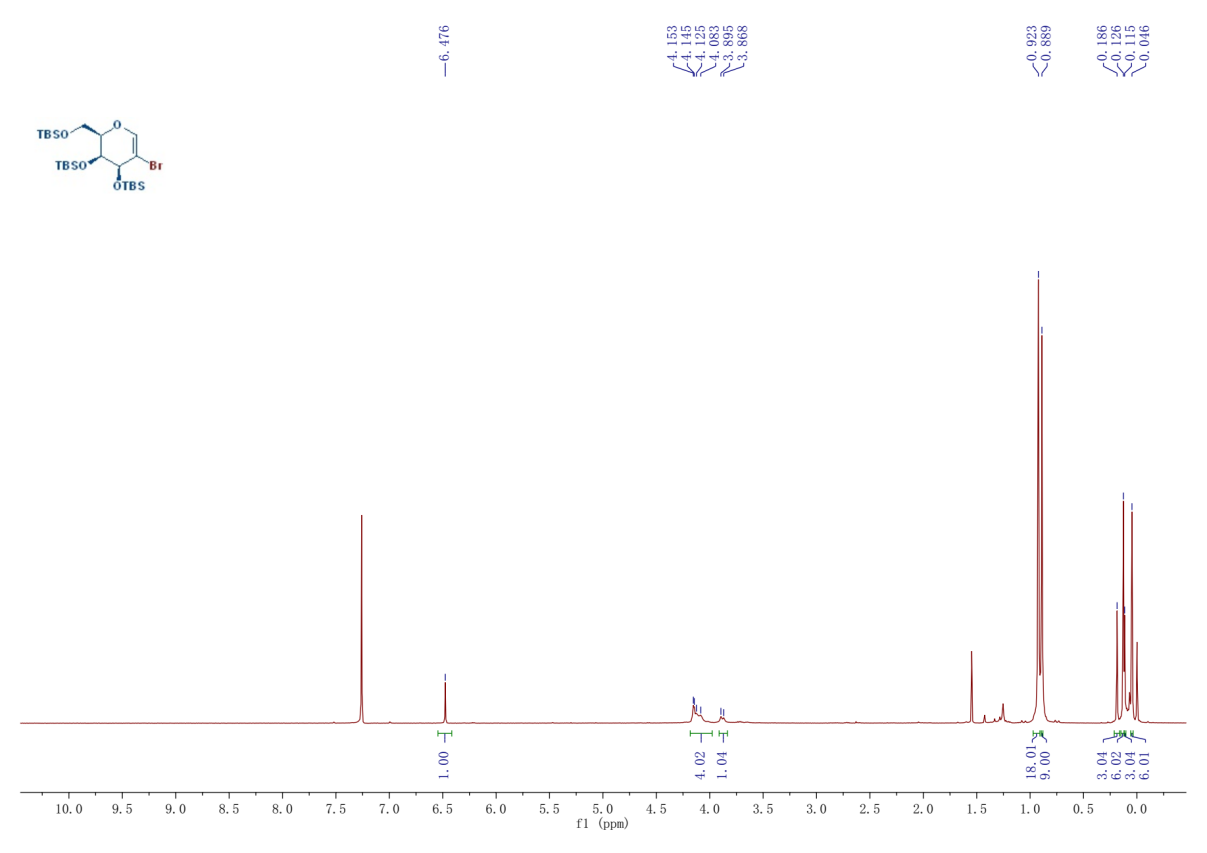


**Supplementary Figure 16. ^1^H NMR spectrum of 3j (400 MHz, CDCl_3_)**


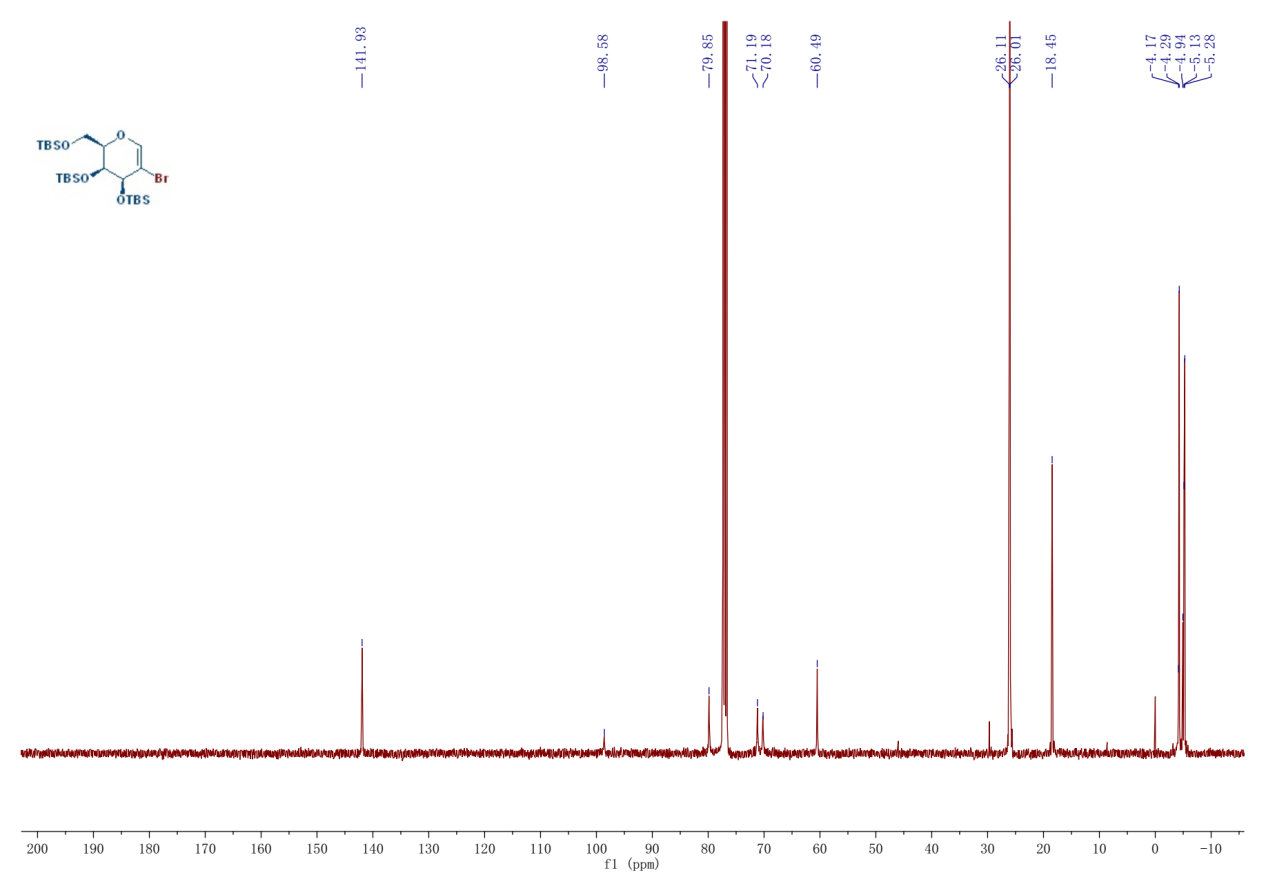


**Supplementary Figure 17. ^13^C NMR spectrum of 3j (101 MHz, CDCl_3_)**


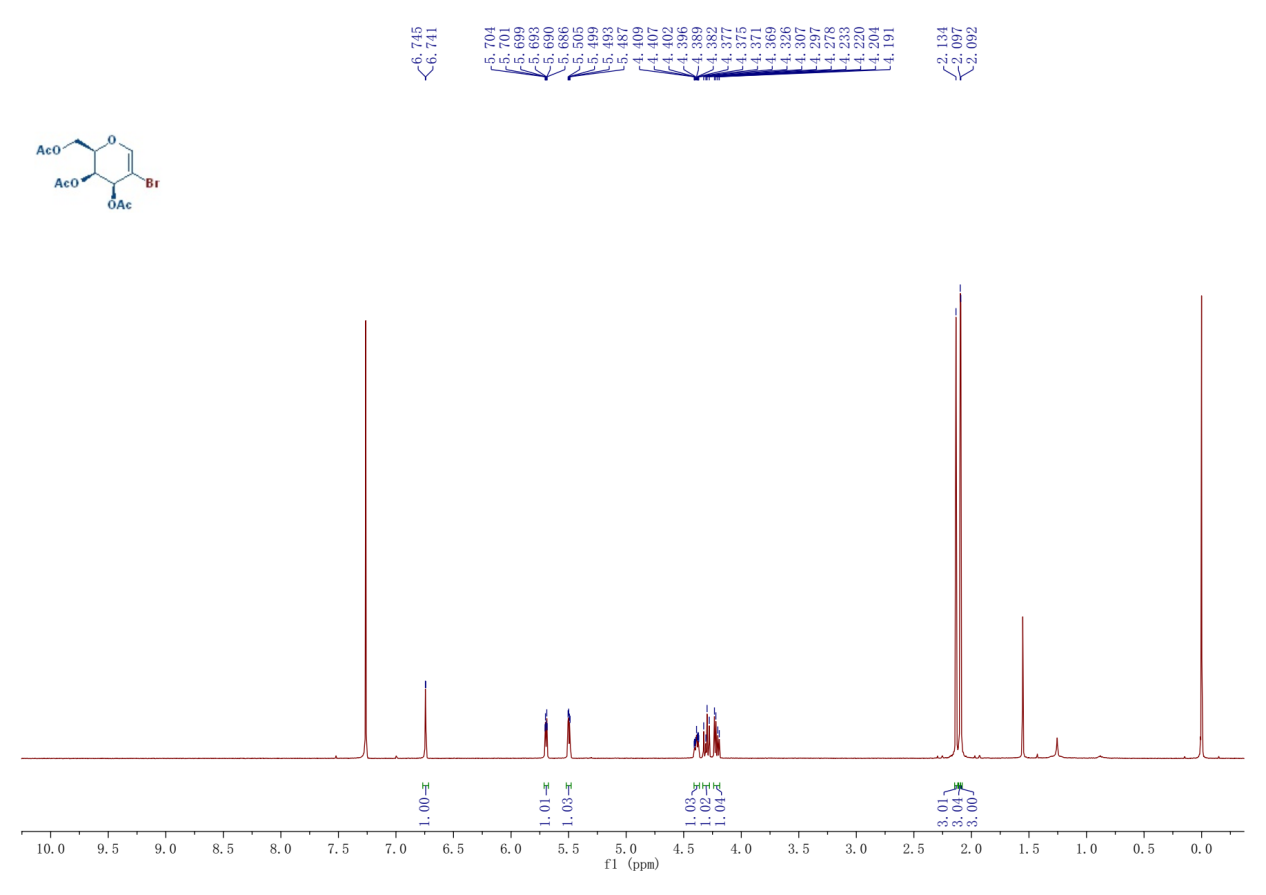


**Supplementary Figure 18. ^1^H NMR spectrum of 3k (400 MHz, CDCl_3_)**


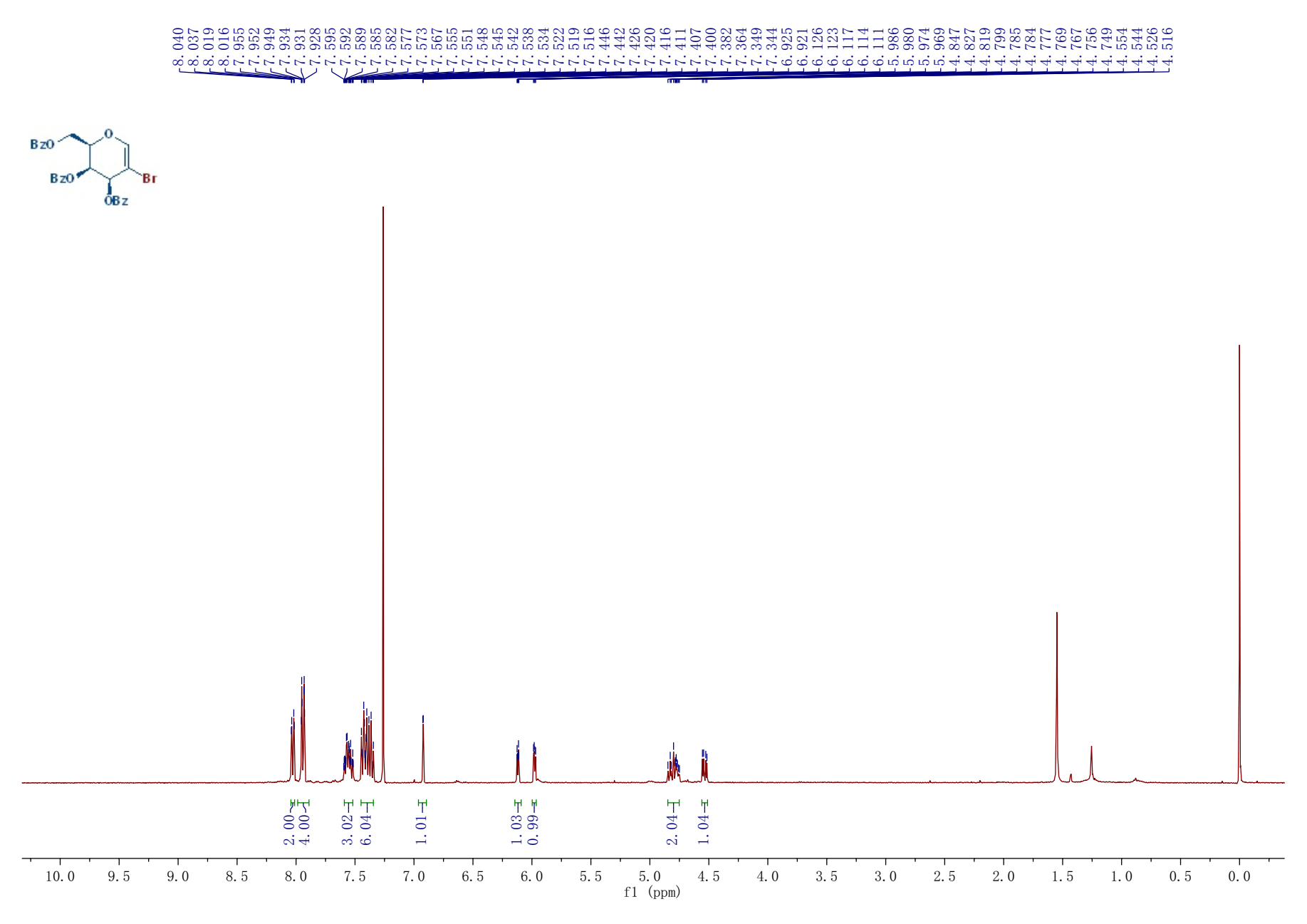


**Supplementary Figure 19. ^1^H NMR spectrum of 3l (400 MHz, CDCl_3_)**


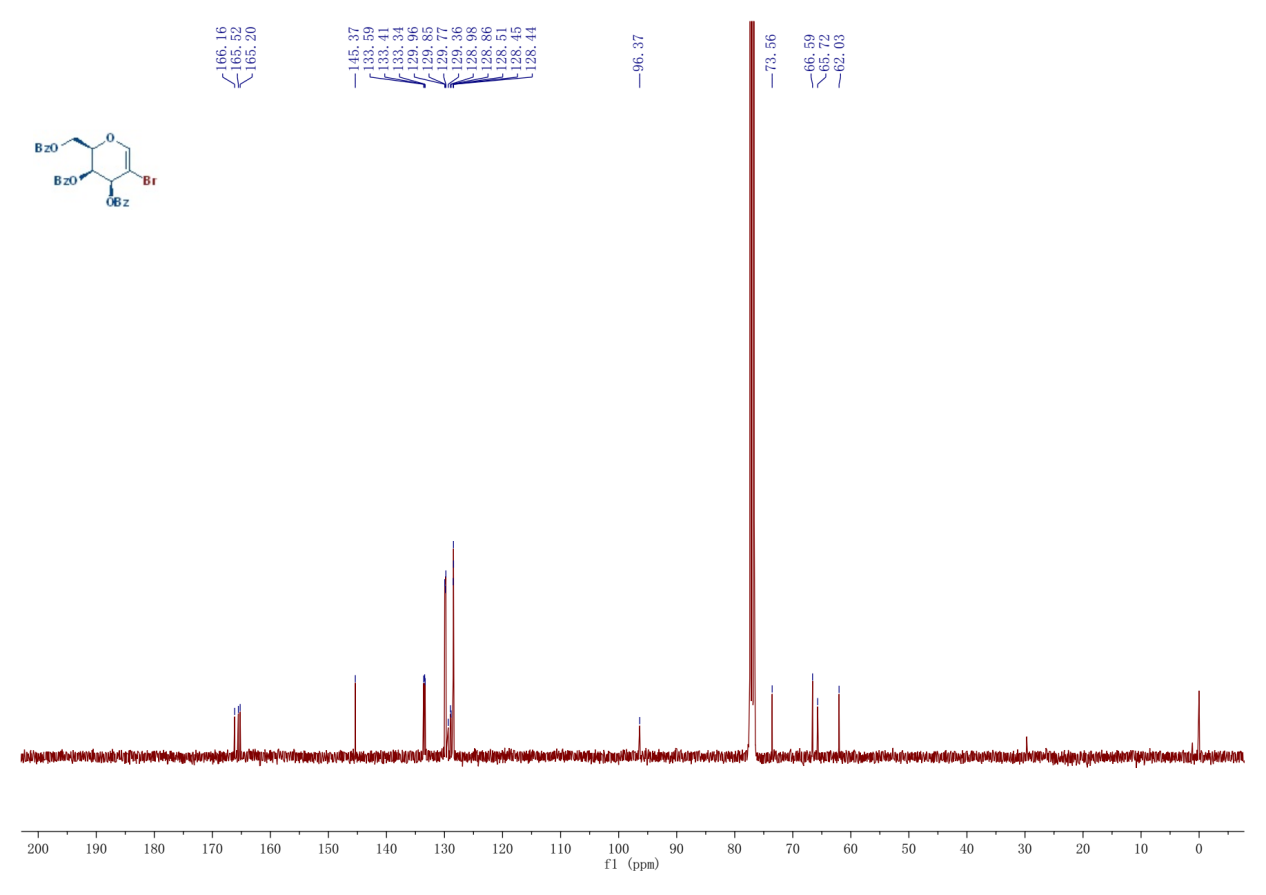


**Supplementary Figure 20. ^13^C NMR spectrum of 3l (101 MHz, CDCl_3_)**


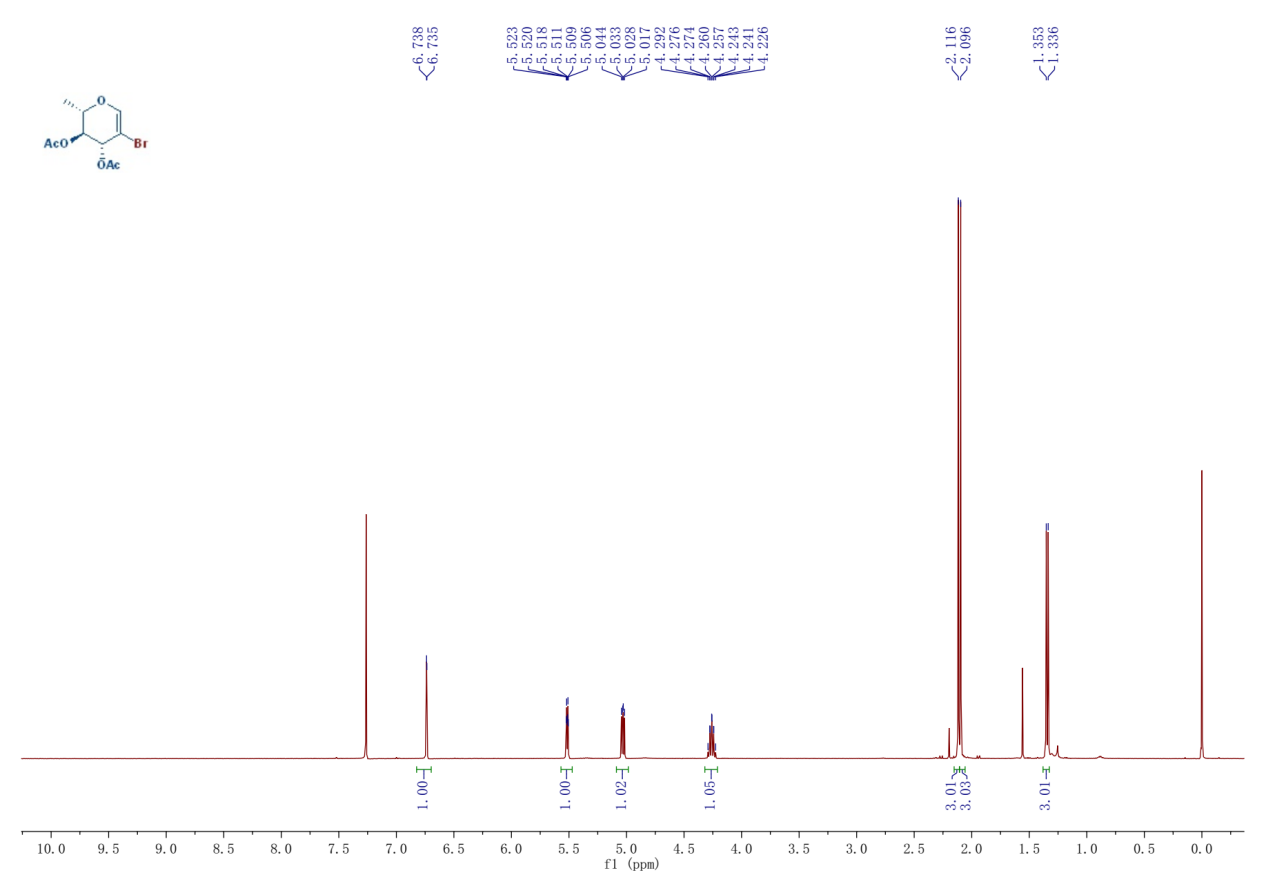


**Supplementary Figure 21. ^1^H NMR spectrum of 3m (400 MHz, CDCl_3_)**


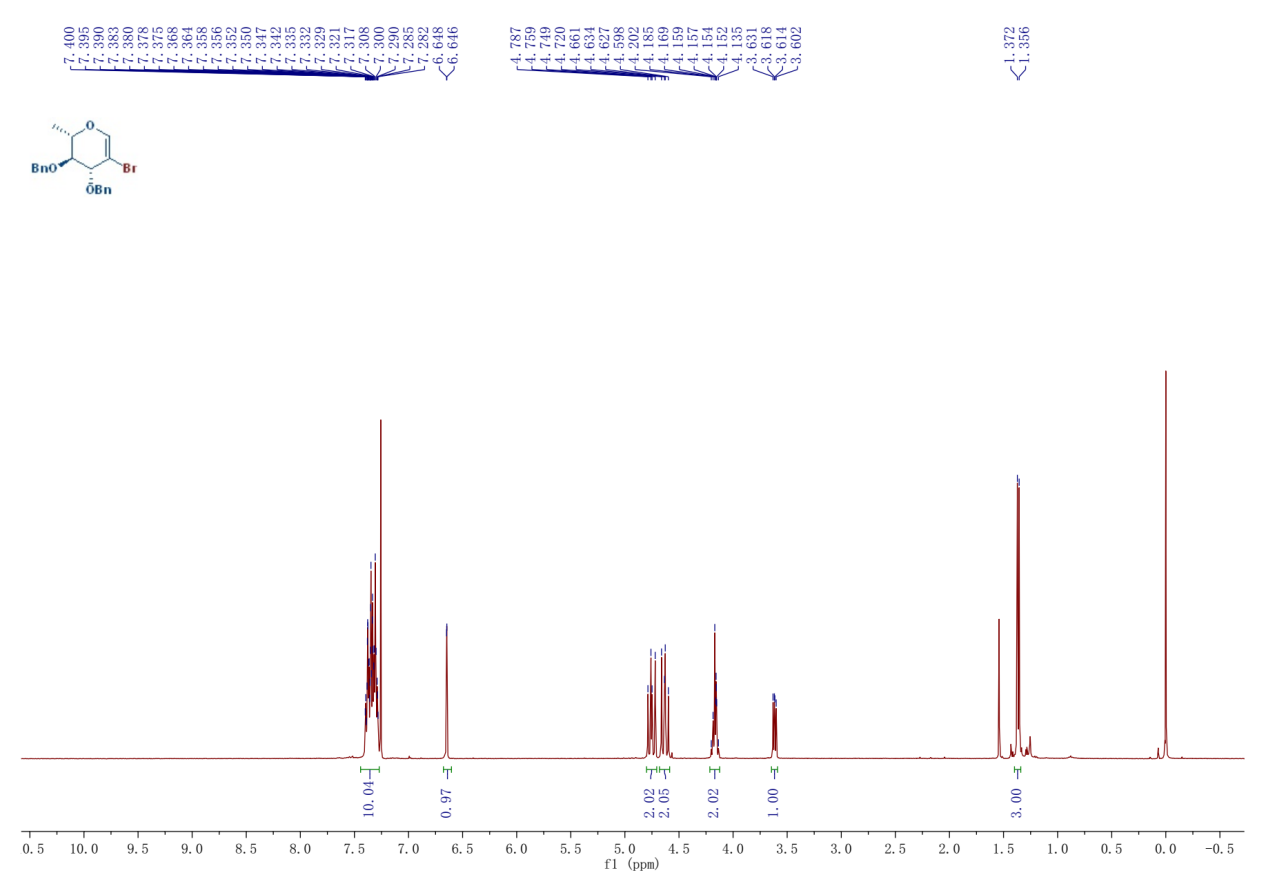


**Supplementary Figure 22. ^1^H NMR spectrum of 3n (400 MHz, CDCl_3_)**


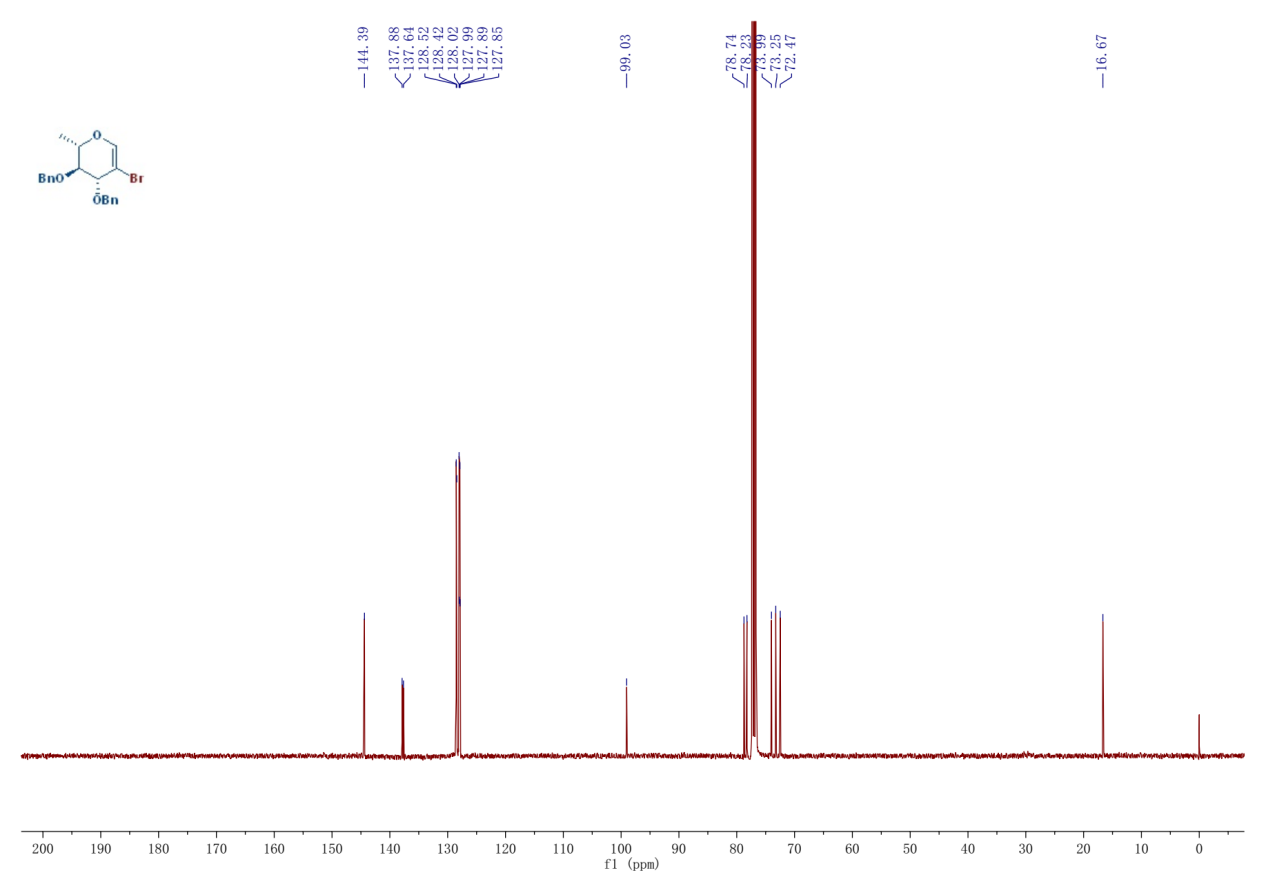


**Supplementary Figure 23. ^13^C NMR spectrum of 3n (101 MHz, CDCl_3_)**


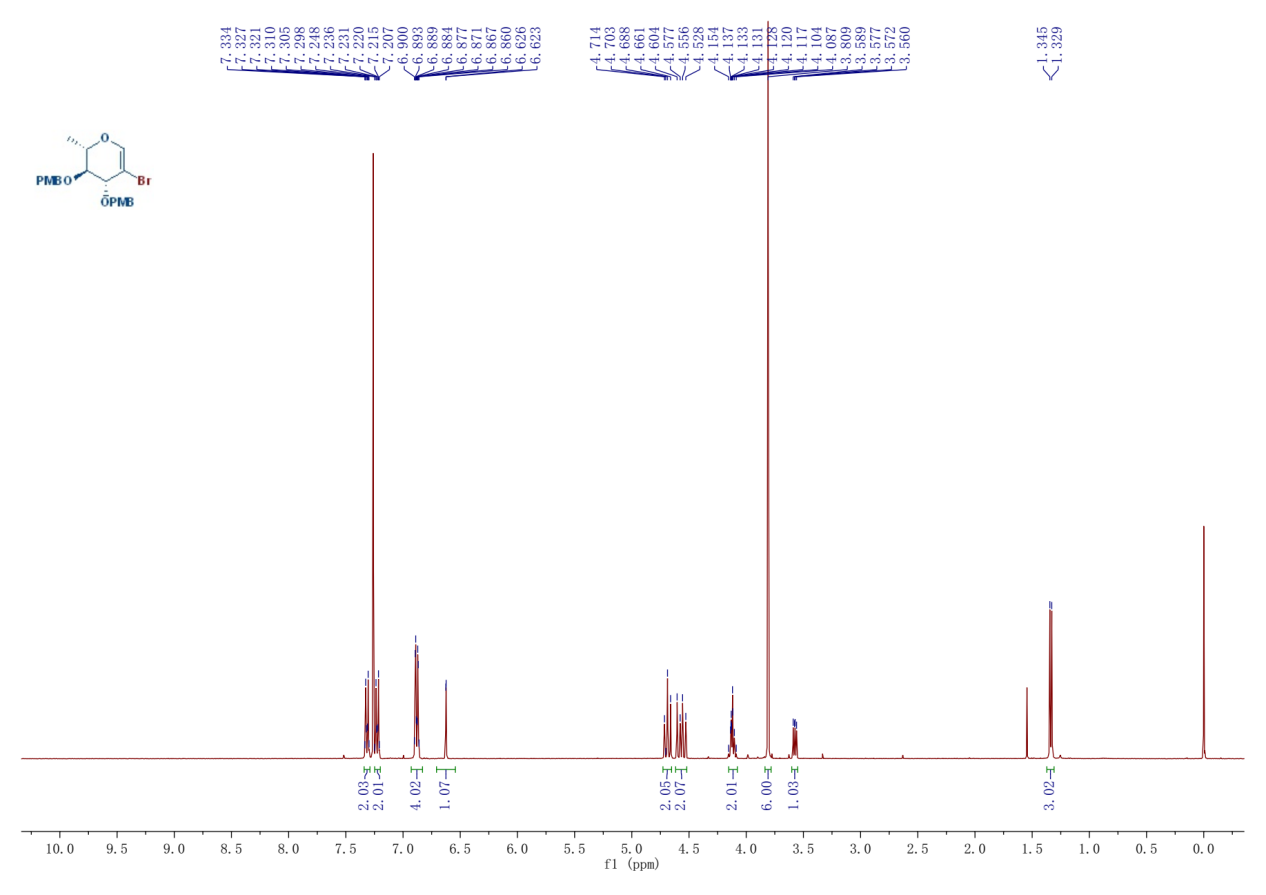


**Supplementary Figure 24. ^1^H NMR spectrum of 3o (400 MHz, CDCl_3_)**


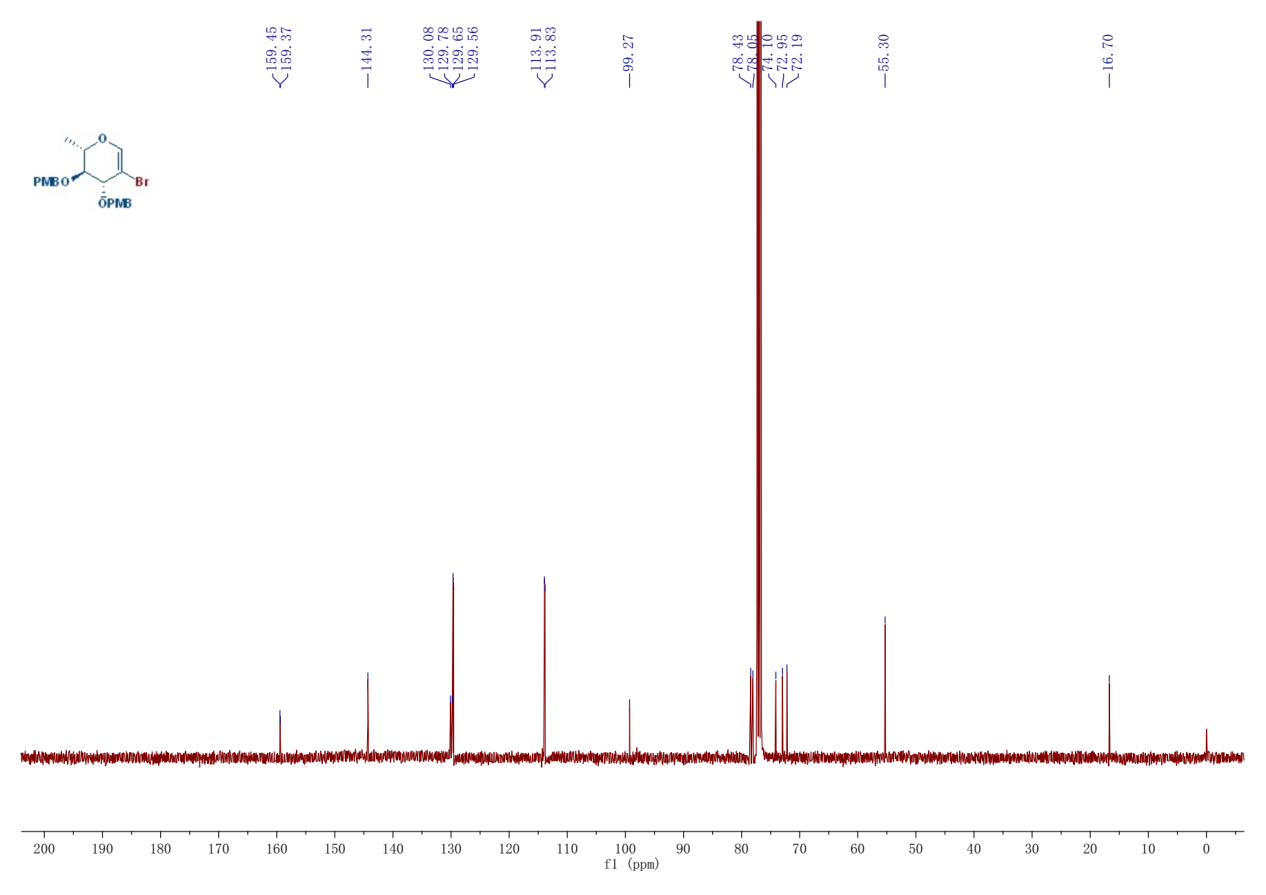


**Supplementary Figure 25. ^13^C NMR spectrum of 3o (101 MHz, CDCl_3_)**


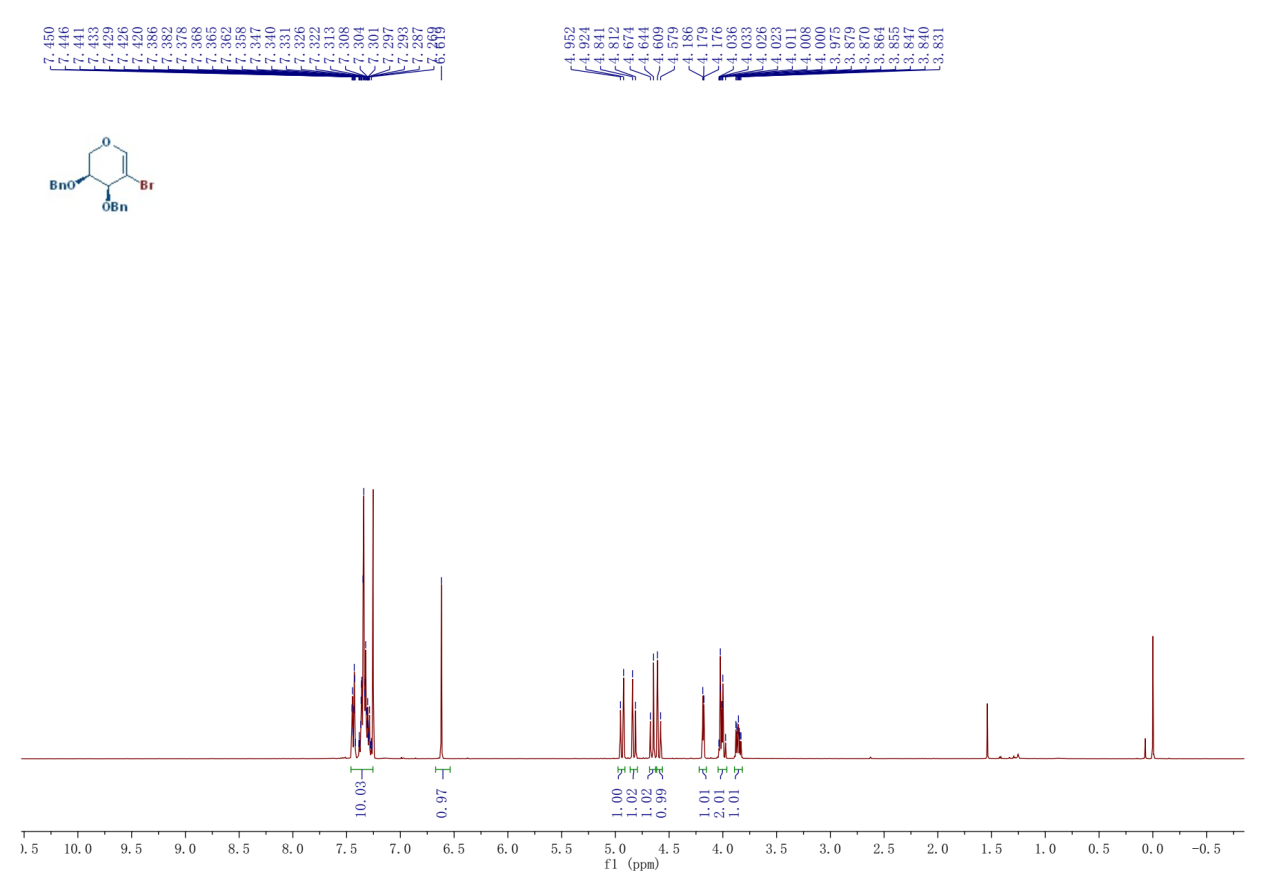


**Supplementary Figure 26. ^1^H NMR spectrum of 3p (400 MHz, CDCl_3_)**


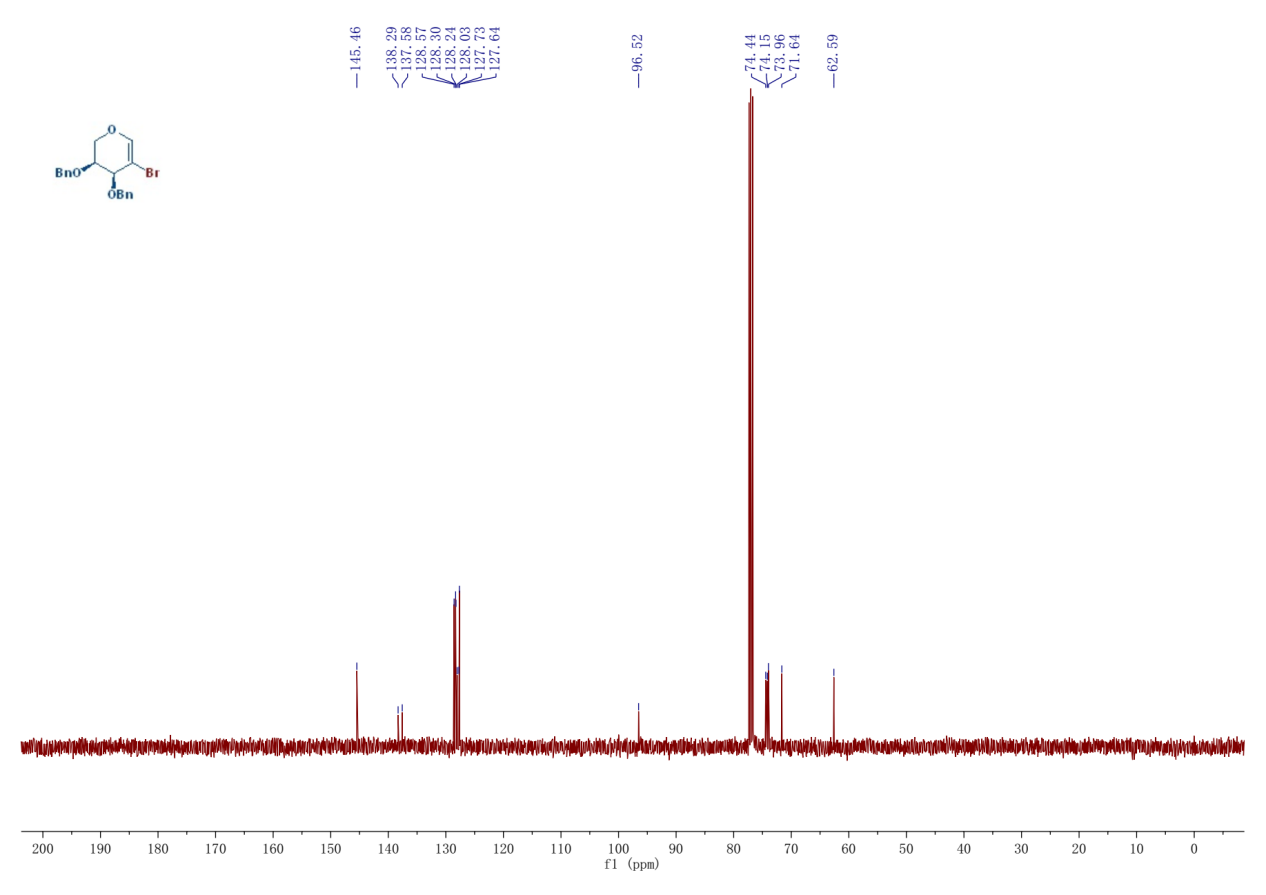


**Supplementary Figure 27. ^13^C NMR spectrum of 3p (101 MHz, CDCl_3_)**


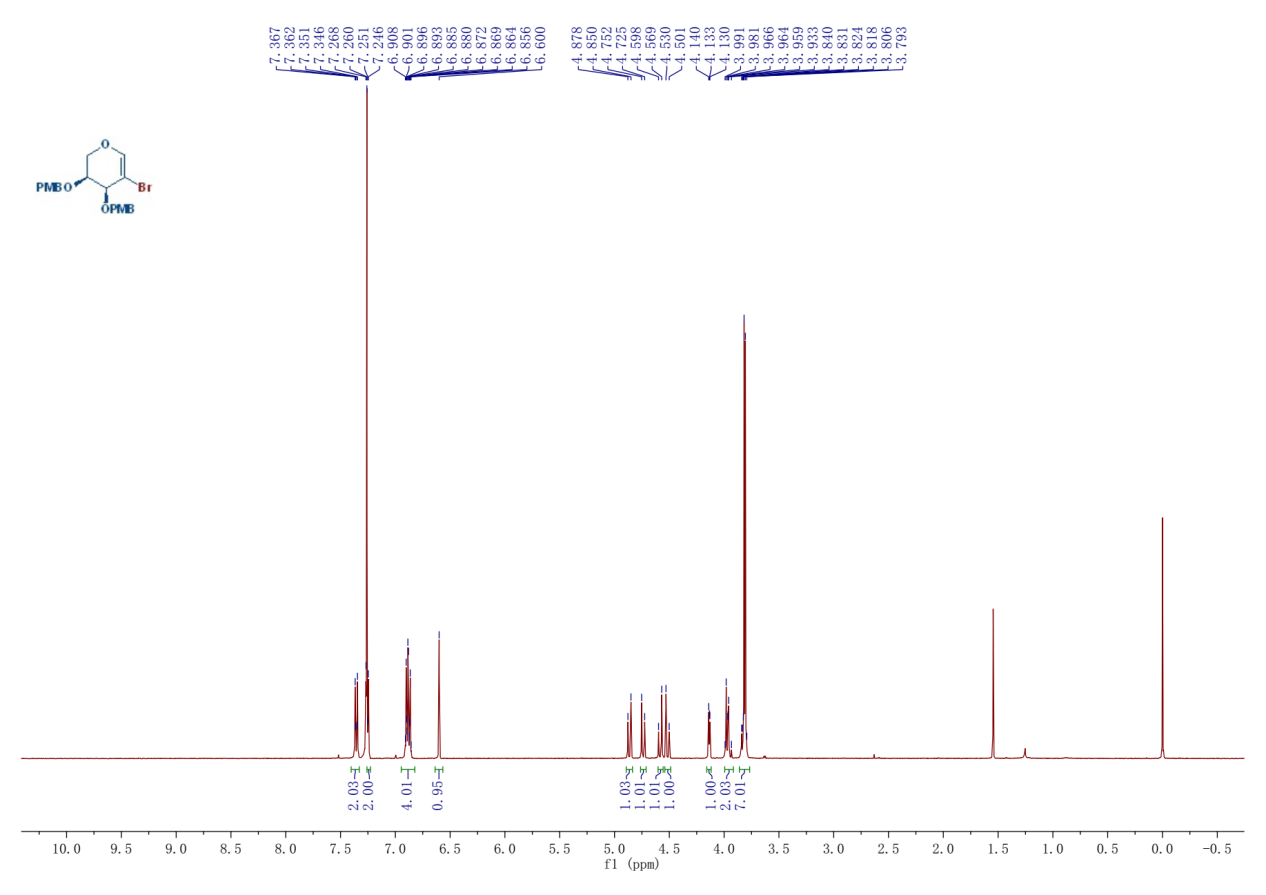


**Supplementary Figure 28. ^1^H NMR spectrum of 3q (400 MHz, CDCl_3_)**


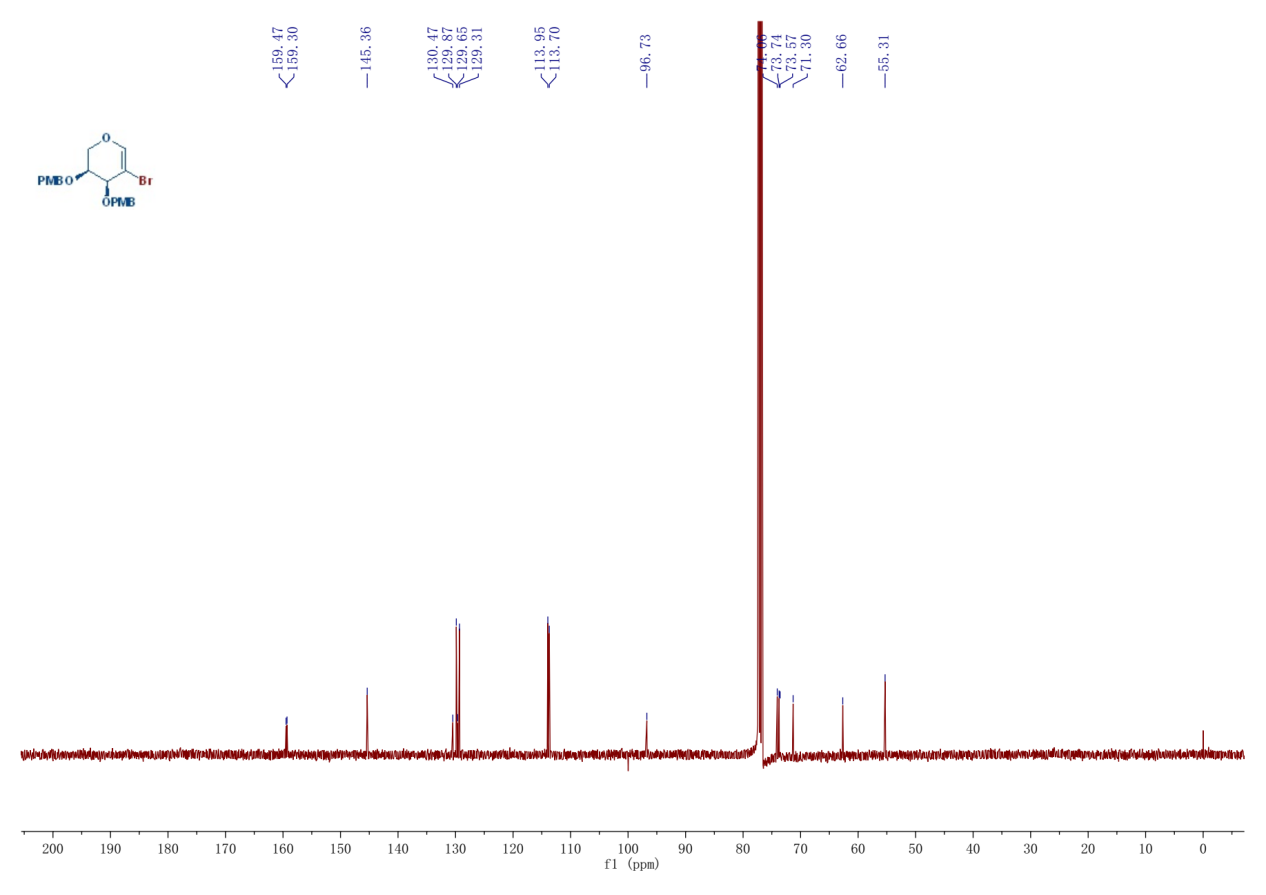


**Supplementary Figure 29. ^13^C NMR spectrum of 3q (101 MHz, CDCl_3_)**


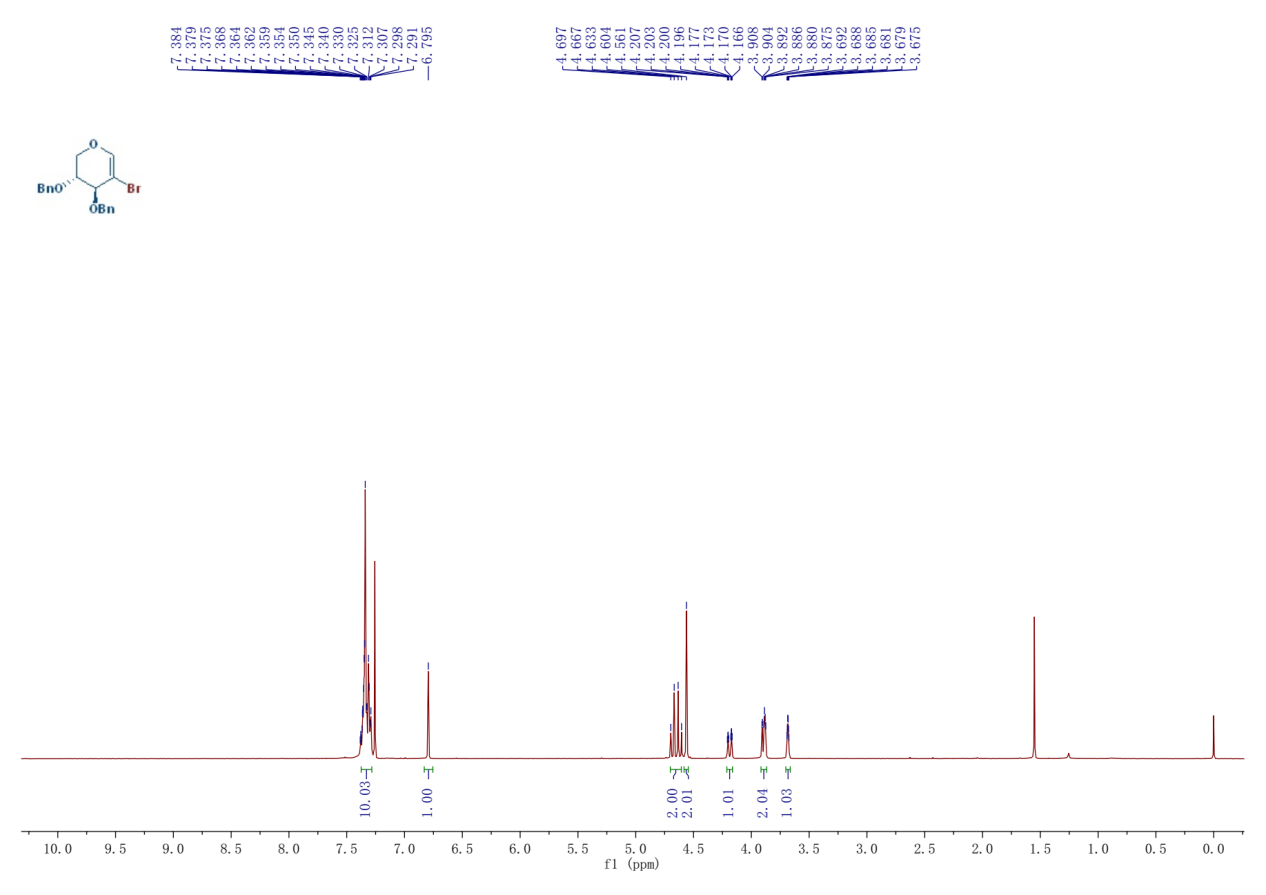


**Supplementary Figure 30. ^1^H NMR spectrum of 3r (400 MHz, CDCl_3_)**


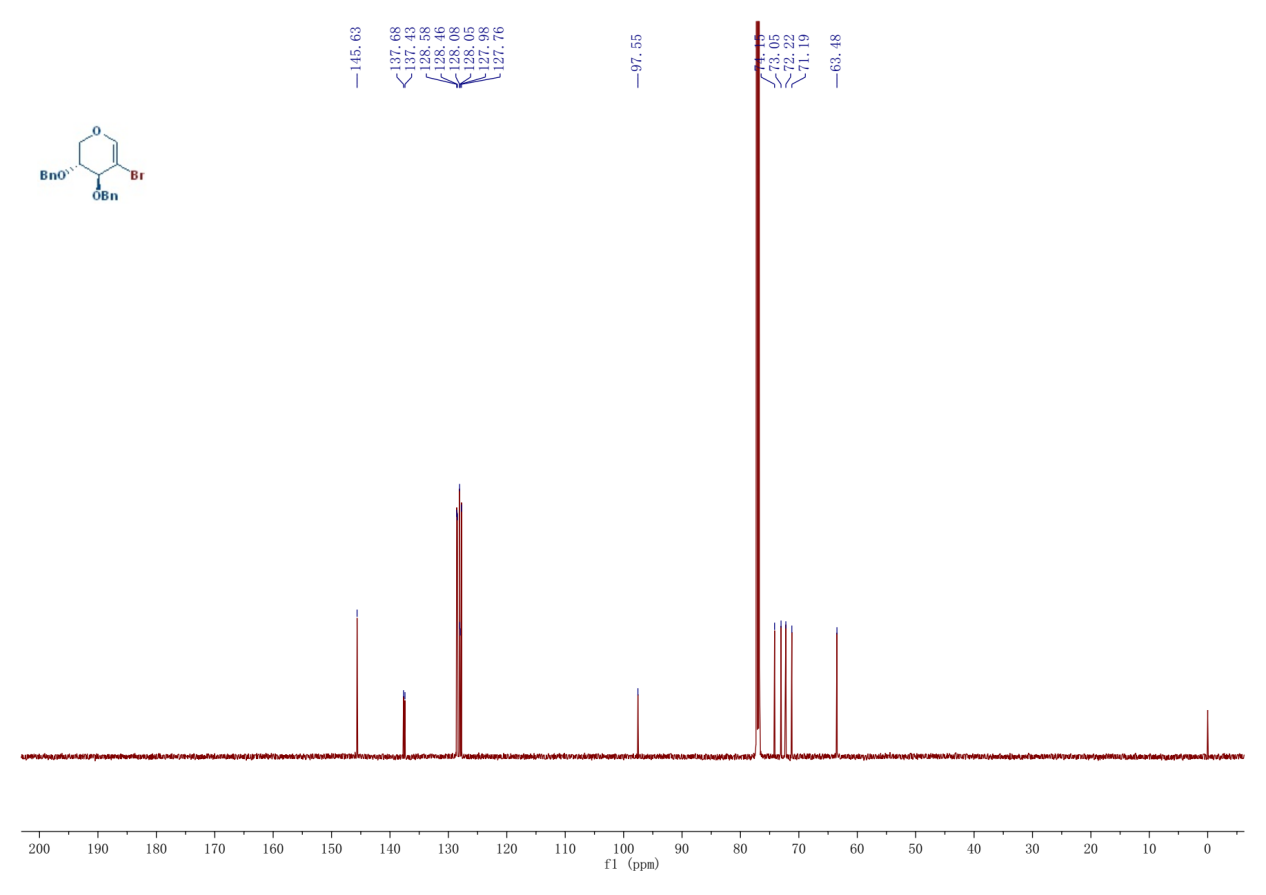


**Supplementary Figure 31. ^13^C NMR spectrum of 3r (151 MHz, CDCl_3_)**


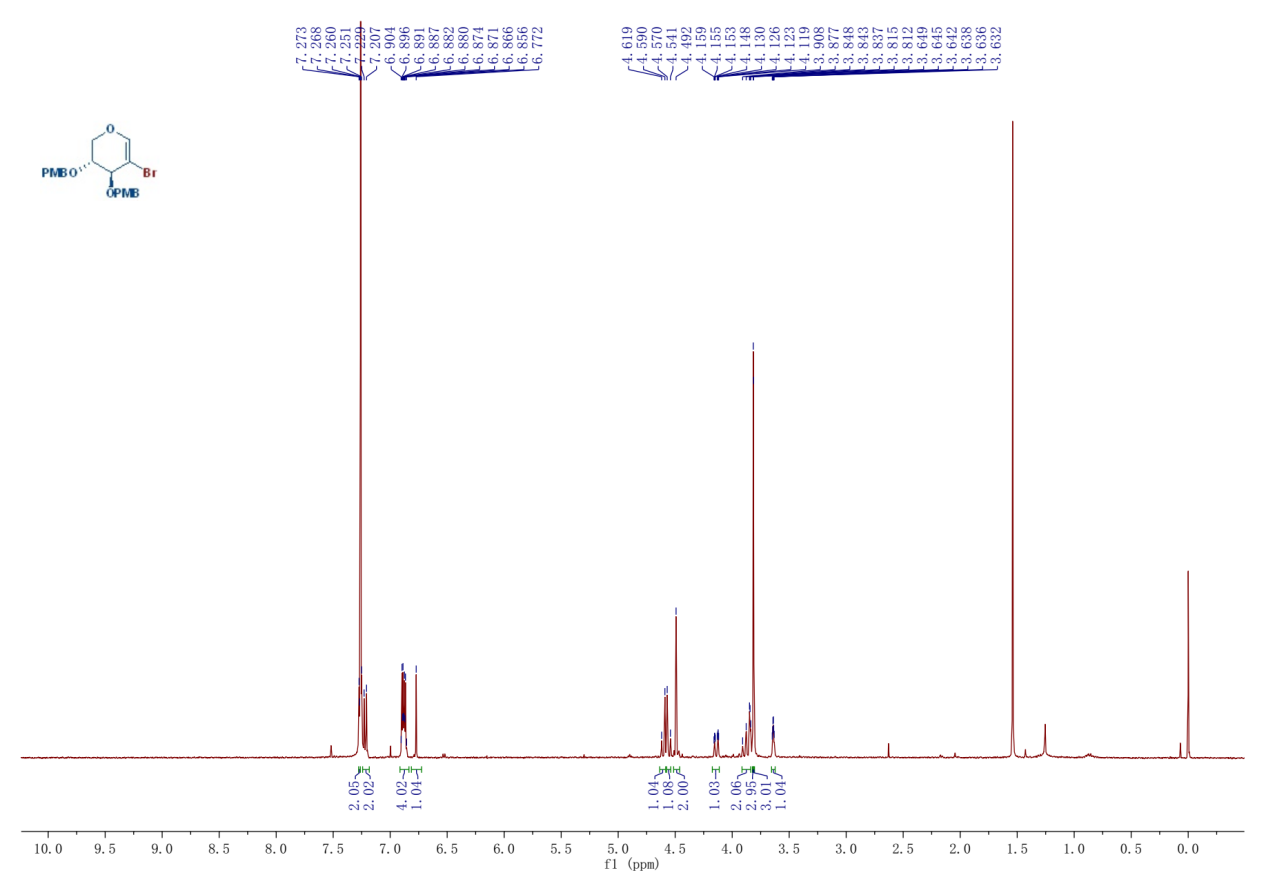


**Supplementary Figure 32. ^1^H NMR spectrum of 3s (400 MHz, CDCl_3_)**


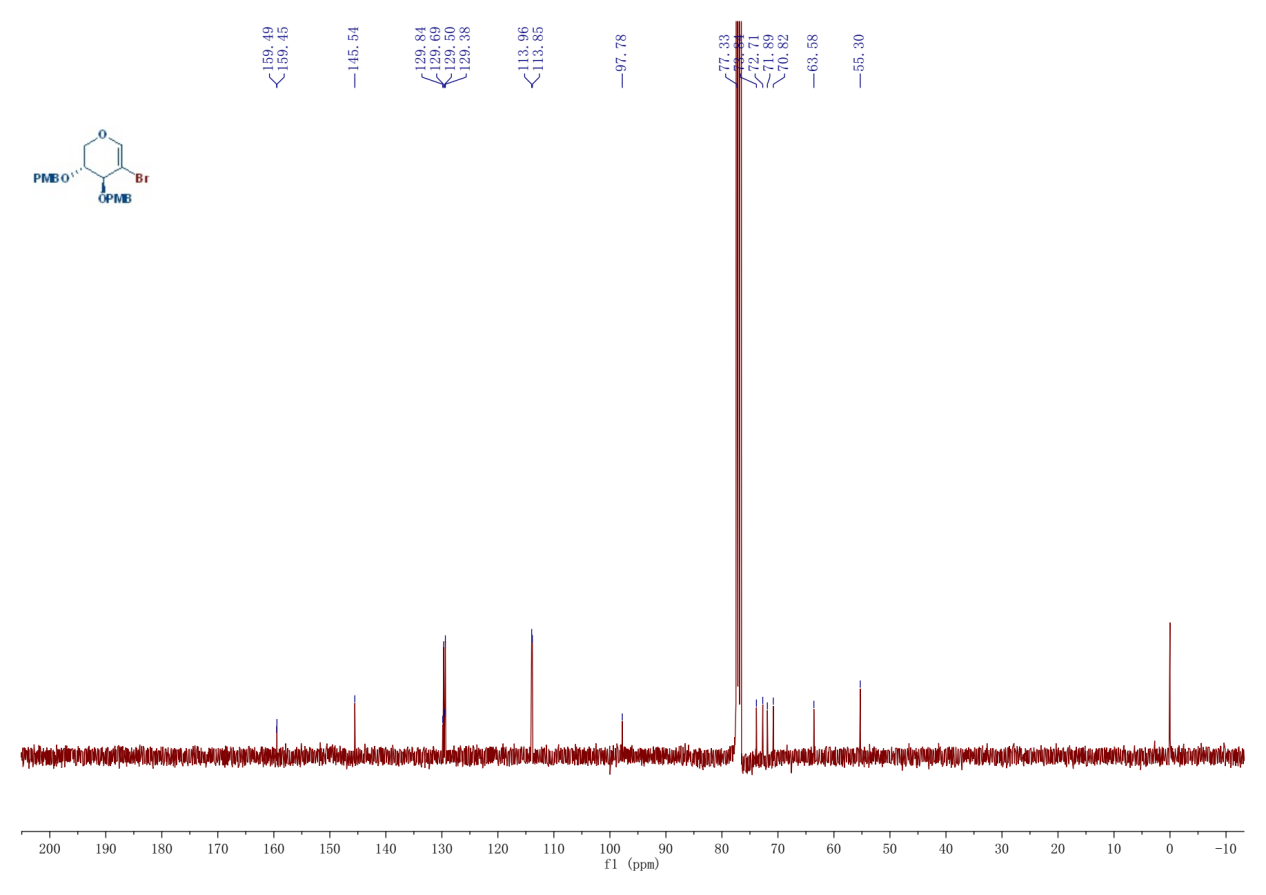


**Supplementary Figure 33. ^13^C NMR spectrum of 3s (101 MHz, CDCl_3_)**


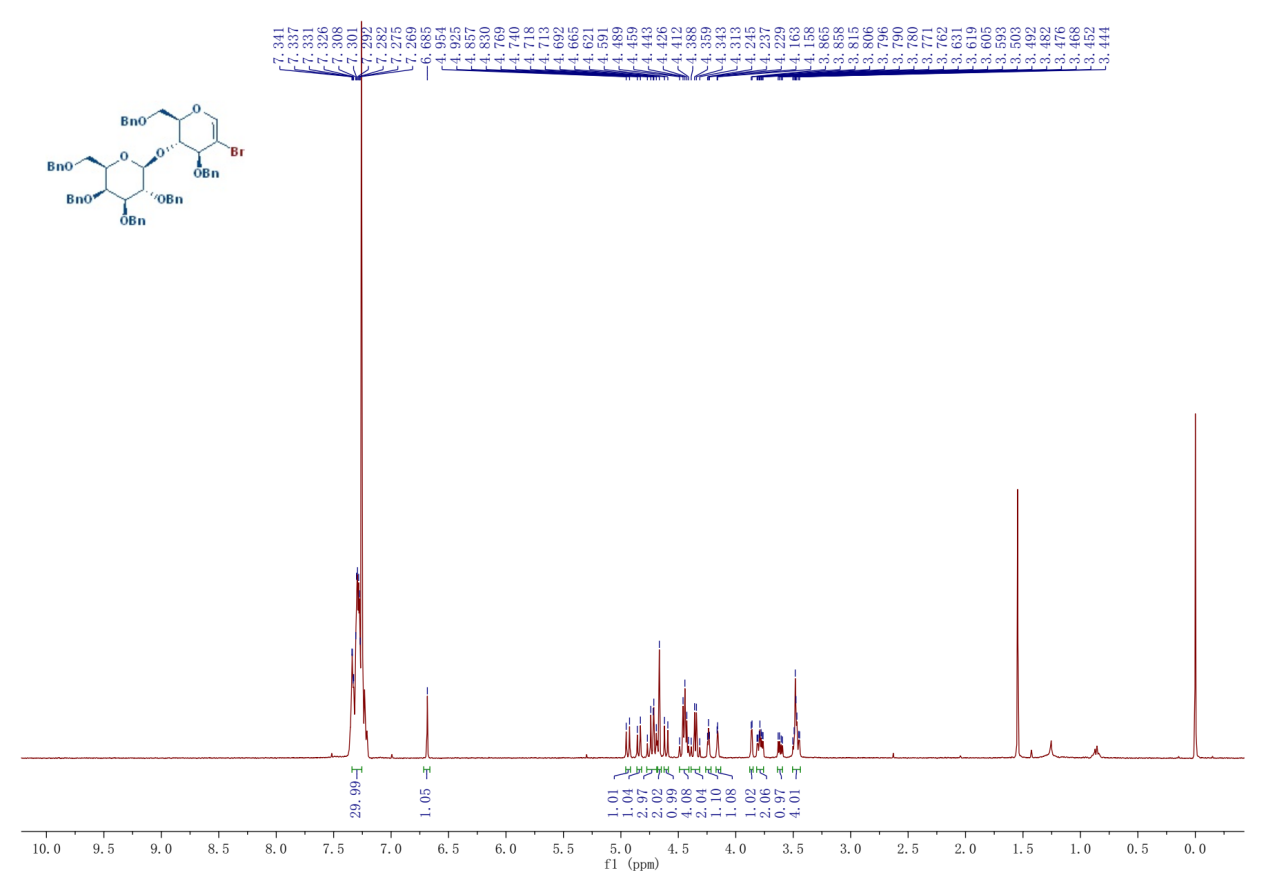


**Supplementary Figure 34. ^1^H NMR spectrum of 3t (400 MHz, CDCl_3_)**


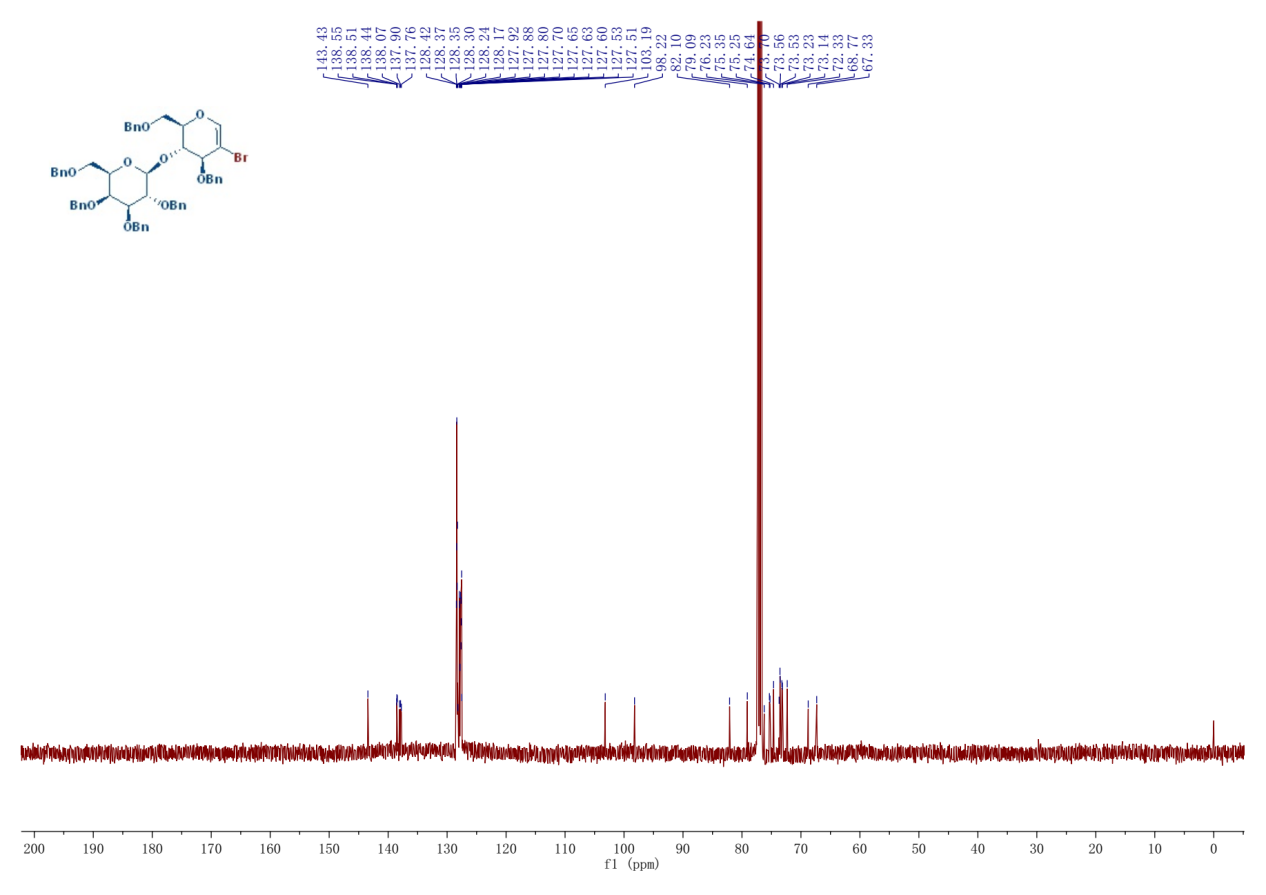


**Supplementary Figure 35. ^13^C NMR spectrum of 3t (101 MHz, CDCl_3_)**


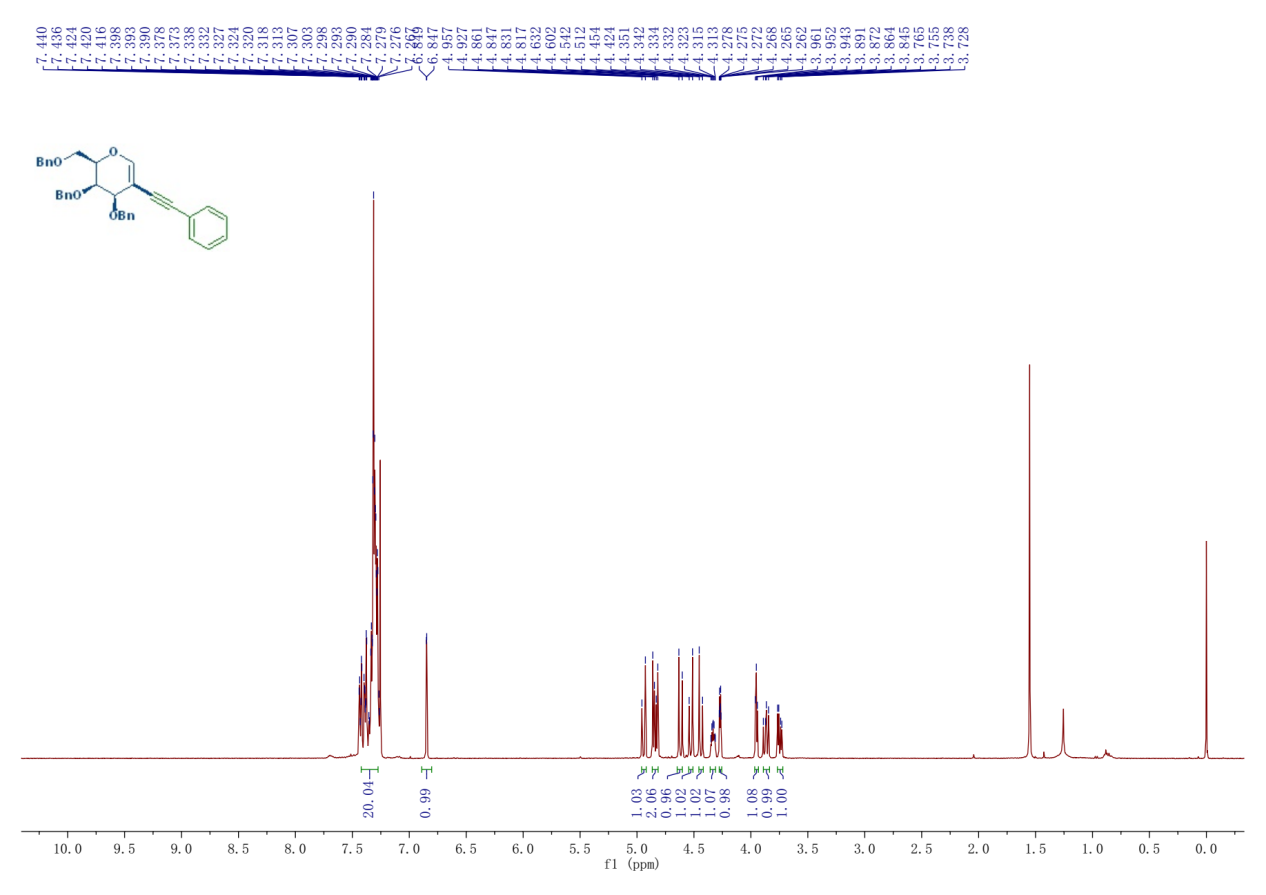


**Supplementary Figure 36. ^1^H NMR spectrum of 5a (400 MHz, CDCl_3_)**


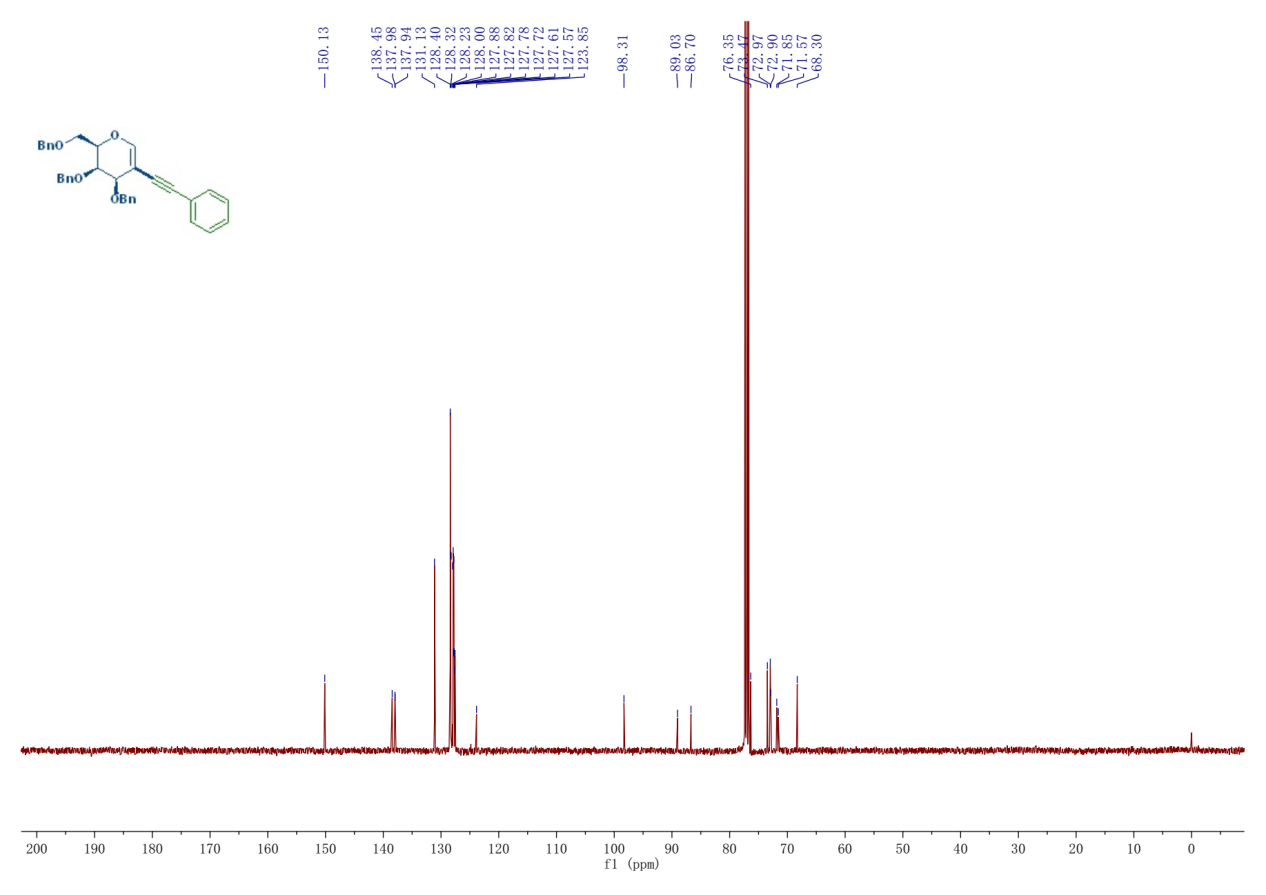


**Supplementary Figure 37. ^13^C NMR spectrum of 5a (101 MHz, CDCl_3_)**


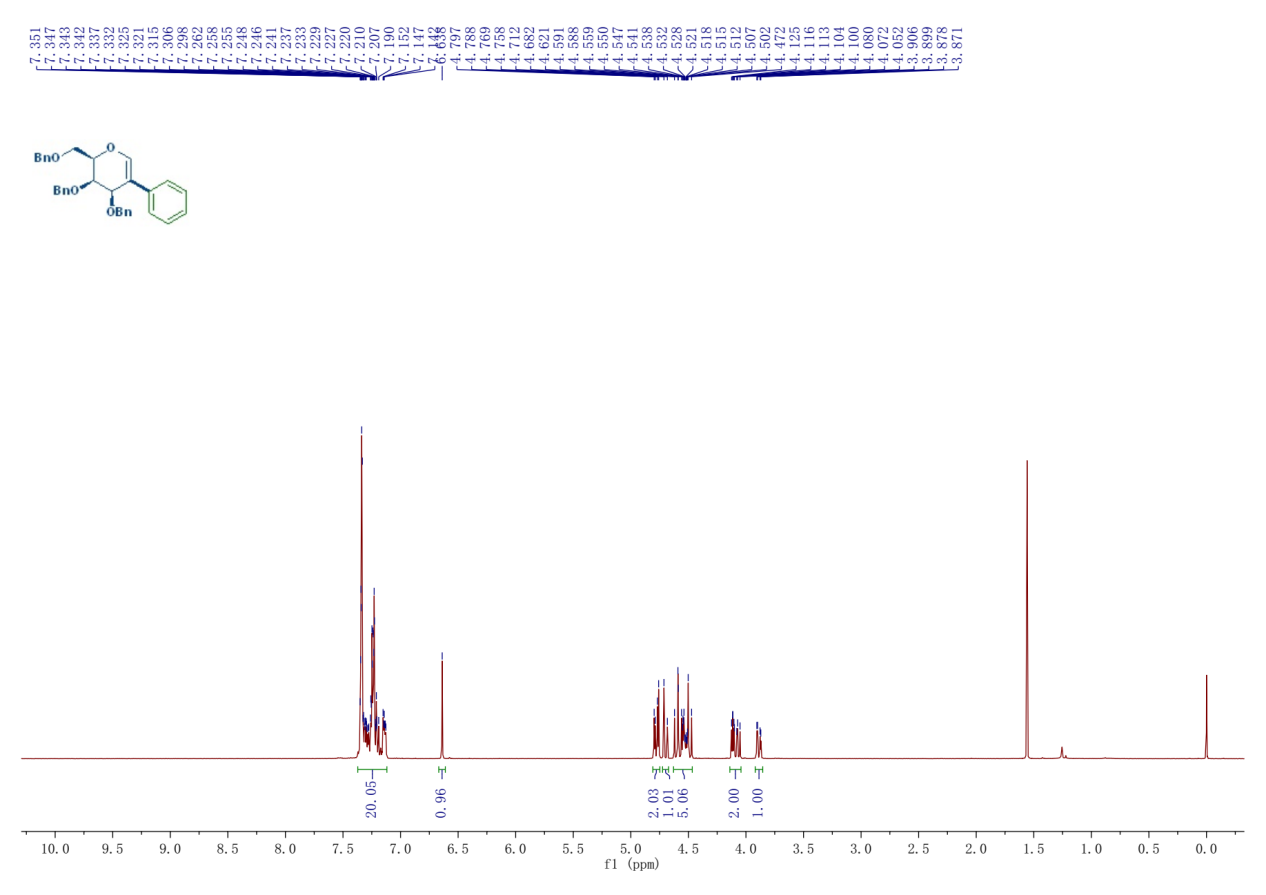


**Supplementary Figure 38. ^1^H NMR spectrum of 5b (400 MHz, CDCl_3_)**


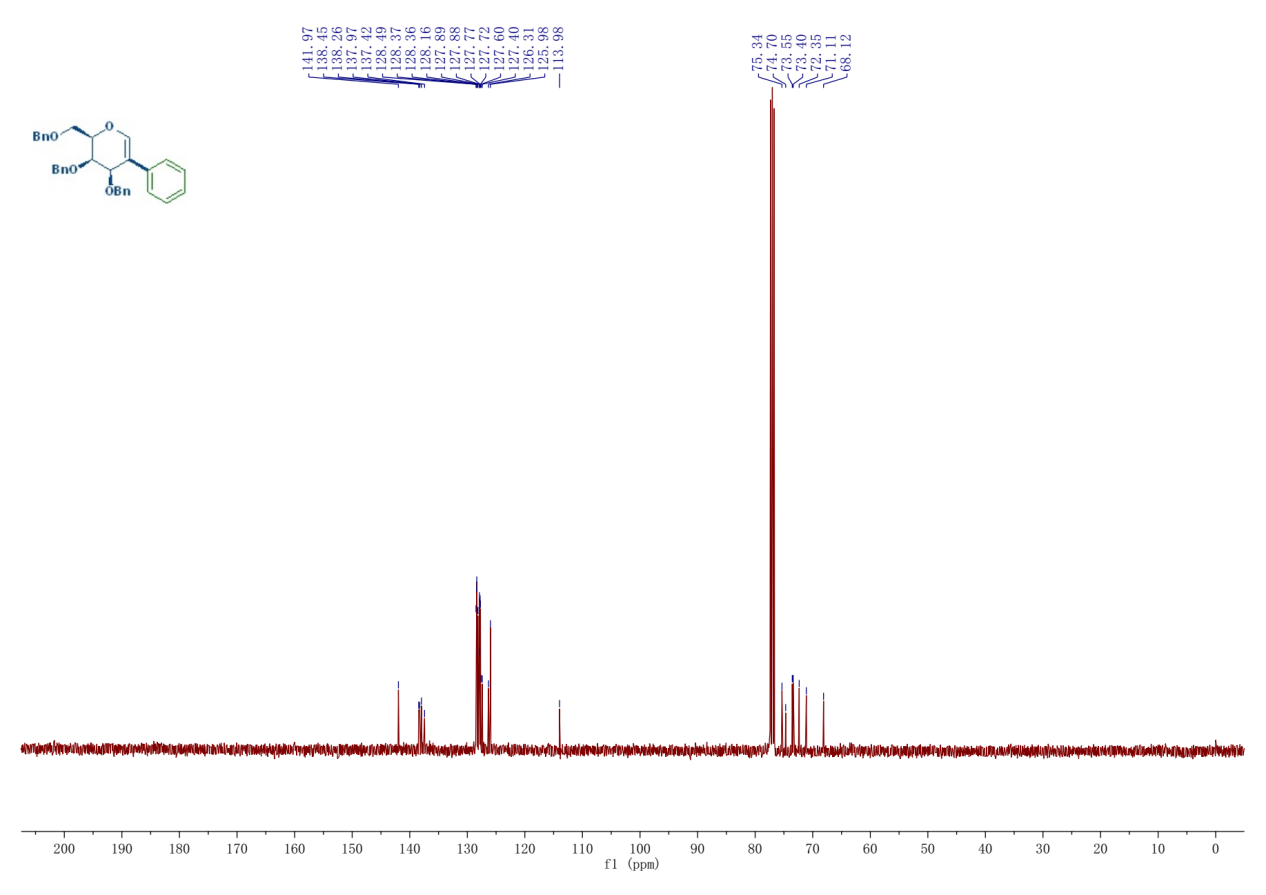


**Supplementary Figure 39. ^13^C NMR spectrum of 5b (101 MHz, CDCl_3_)**


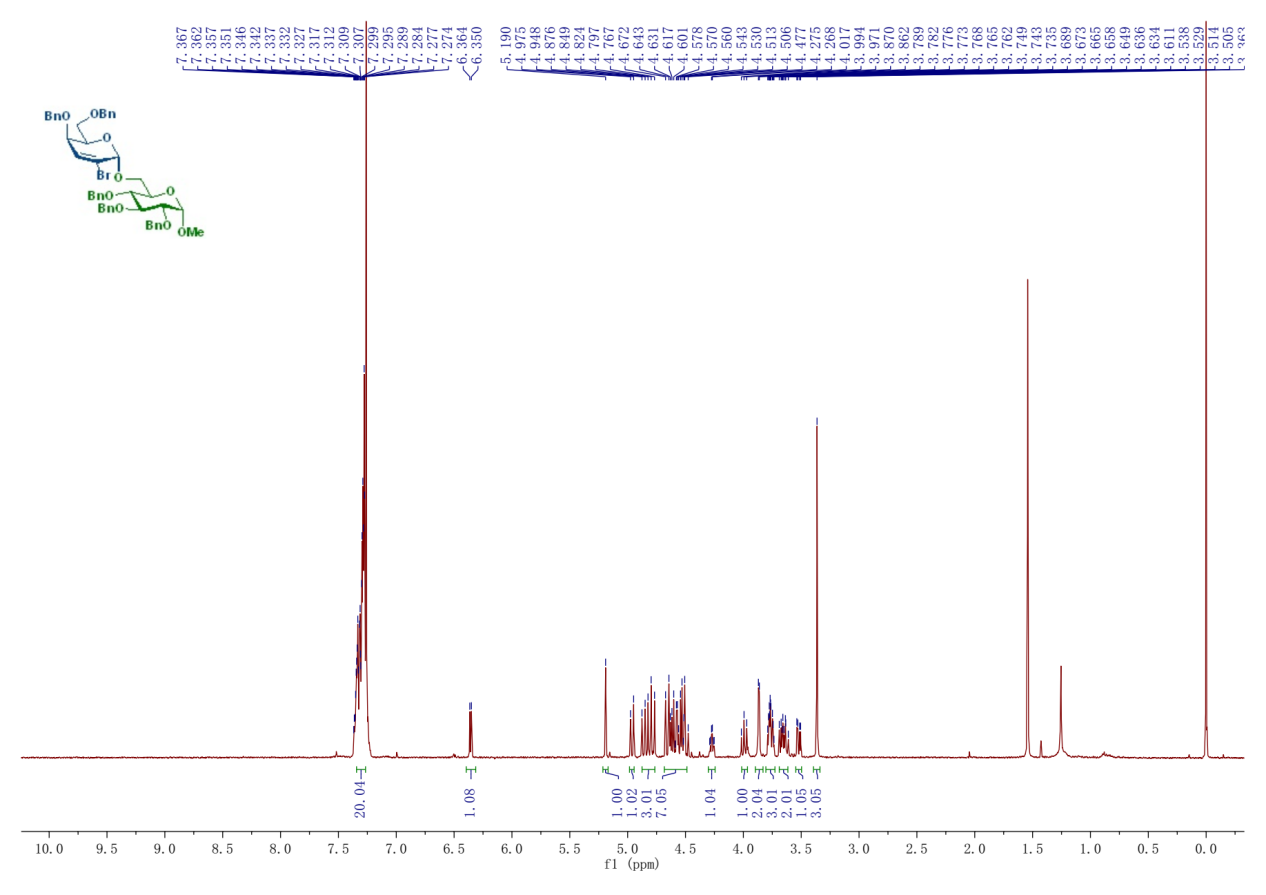


**Supplementary Figure 40. ^1^H NMR spectrum of 5c (400 MHz, CDCl_3_)**


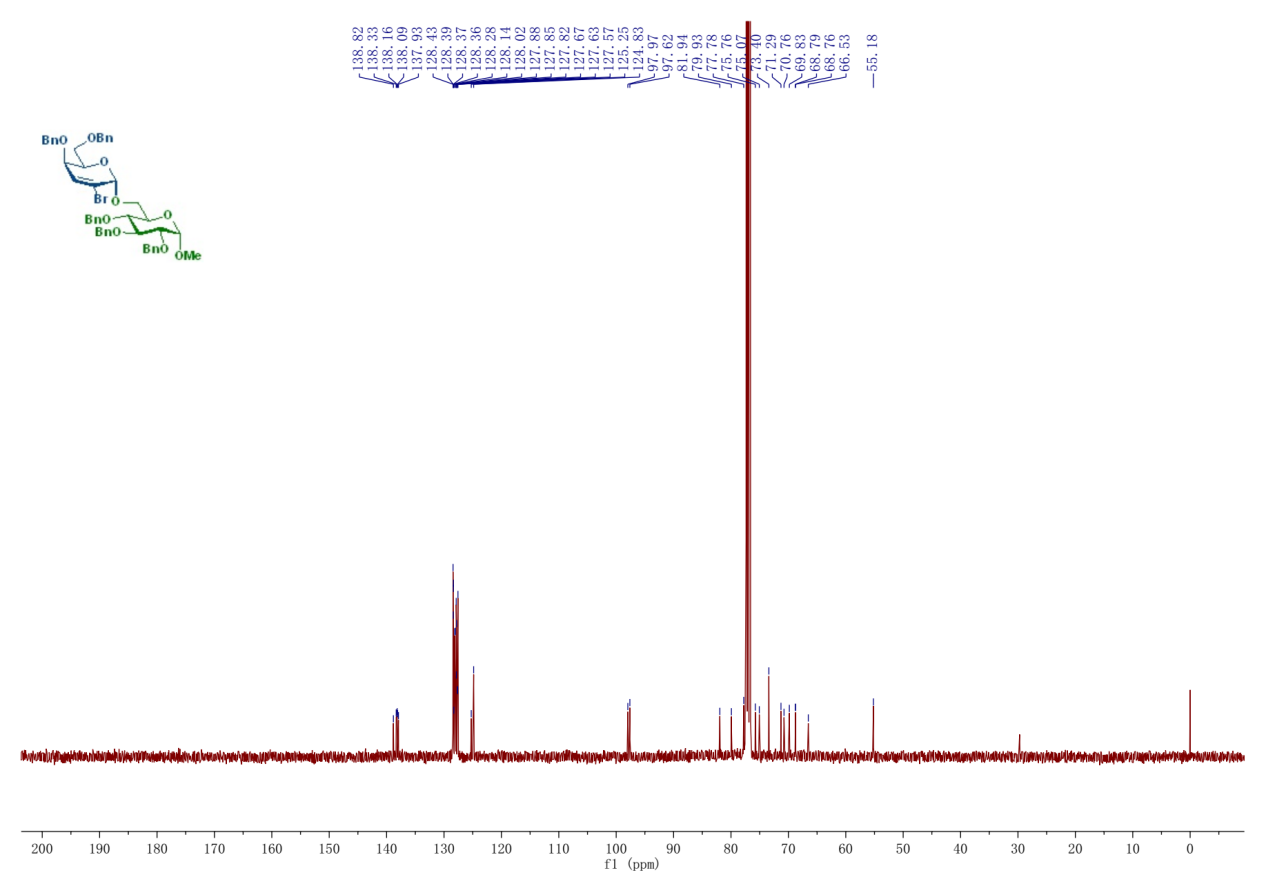


**Supplementary Figure 41. ^13^C NMR spectrum of 5c (101 MHz, CDCl_3_)**
